# A Blood-Based Assay for Detection of Patients with Advanced Adenomas

**DOI:** 10.1158/2767-9764.CRC-24-0398

**Published:** 2025-04-16

**Authors:** Kamel Lahouel, Christopher Douville, Brenda Diergaarde, Joshua D. Cohen, Haley Grant, Albert Kuo, Saad K. Ansari, Yuxuan Wang, Anne Marie O’Broin-Lennon, Maria Popoli, Janine Ptak, Natalie Silliman, Lisa Dobbyn, Nadine Nehme, Jeanne Tie, Peter Gibbs, Nickolas Papadopoulos, Kenneth W. Kinzler, Bert Vogelstein, Robert E. Schoen, Cristian Tomasetti

**Affiliations:** 1 Center for Cancer Prevention and Early Detection, City of Hope, Duarte, California.; 2 Division of Mathematics for Cancer Evolution and Early Detection, Department of Computational and Quantitative Medicine, Beckman Research Institute, City of Hope, Duarte, California.; 3 Division of Integrated Cancer Genomics, Translational Genomics Research Institute, Phoenix, Arizona.; 4 Ludwig Center for Cancer Genetics and Therapeutics, Johns Hopkins University School of Medicine, Baltimore, Maryland.; 5 Department of Human Genetics, School of Public Health, University of Pittsburgh, Pittsburgh, Pennsylvania.; 6 Department of Biostatistics, Johns Hopkins University School of Public Health, Baltimore, Maryland.; 7 Department of Applied Mathematics and Statistics, Johns Hopkins University, Baltimore, Maryland.; 8 Division of Personalized Oncology, Walter and Eliza Hall Institute of Medical Research, Melbourne, Australia.; 9 Department of Medicine, University of Pittsburgh, Pittsburgh, Pennsylvania.; 10 Department of Epidemiology, School of Public Health, University of Pittsburgh, Pittsburgh, Pennsylvania.

## Abstract

Screening for colorectal cancer with blood-based testing should detect advanced adenomas (AA), facilitating more effective cancer prevention. We evaluated four different methods to detect AAs in plasma: (i) a machine learning algorithm, *Signa*tures of fragment *L*ength (SignaL), based on cell-free DNA (cfDNA) fragmentation; (ii) a “Protein-17” assay measuring 17 cancer-associated proteins; (iii) a global aneuploidy score (GAS); and (iv) cfDNA mutation analysis querying 15 genes commonly mutated in colorectal cancer. Existing data from study populations with and without cancer were utilized to determine 99.5% specificity thresholds. We studied 40 AA cases and 32 colonoscopy-negative controls. SignaL detected 9/40 AAs [22.5%, 95% confidence interval (CI), 12.3%–37.5%] at 100% specificity (95% CI, 89.3%–100%). Protein-17 detected 5/40 AAs (12.5%, 95% CI, 5.5%–26.1%) including three cases not identified by SignaL, at 100% specificity (95% CI, 89.3%–100%). GAS detected 11/40 AAs (27.5%, 95% CI, 16.1%–42.8%) but resulted in 2/32 positive controls (93.8% specificity, 95% CI, 79.9%–98.3%). Combining SignaL, the Protein-17 assay, and GAS at the 99.5% specificity thresholds resulted in detection of 16/40 AAs (40%, 95% CI, 26.3%–55.4%) at 93.8% specificity (95% CI, 79.9%–98.3%). Of 32/40 evaluable AA plasma samples, only one was cfDNA mutation positive at level of significance of 0.01. A blood-based assay based on the analysis of repeated sequence elements plus proteins seems to be able to detect a considerable fraction of patients with AA at relatively high specificity. Large, prospective studies are required to determine whether this approach can add to the options currently available for screening patients for premalignant lesions of the colon.

**Significance:**

Blood-based screening for colorectal cancer could improve testing uptake and outcomes. We propose novel methods to detect AAs in plasma using cfDNA fragmentation patterns, cancer-associated proteins, and aneuploidy with high specificity. Larger studies are needed to validate clinical utility.

## Introduction

Colorectal cancer is the fourth most common cancer and the second leading cause of cancer-related death in the United States (1). Screening for colorectal cancer results in a significant reduction in colorectal cancer incidence and mortality. Randomized trials of screening with flexible sigmoidoscopy demonstrate that removing adenomatous polyps prevents subsequent incident colorectal cancer (2, 3). Reducing cancer incidence by detecting and removing precancerous lesions has an even greater impact on cancer mortality than early detection of cancerous lesions, as estimates suggest that reducing cancer incidence accounts for 2/3% to 80% of the mortality reduction resulting from screening (4, 5).

Screening for colorectal cancer is currently performed with stool-based or endoscopic testing (6). There is considerable interest in developing blood-based screening because of the ease with which blood can be sampled. This could potentially increase the population uptake of colorectal cancer screening from its current level of compliance of only 68% (7) in the United States. It would be optimal for blood-based screening tests to be able to detect not only early stage for colorectal cancer but also for premalignant lesions which are most likely to evolve into cancer, such as advanced adenomas (AA). Patients with AAs are at a three-fold long-term increased risk of colorectal cancer compared with those without adenomas (8–10). Stool-based testing such as fecal immunochemical test (FIT) or multi-target stool DNA (mt-sDNA) detects 25% to 42% of AAs (11, 12) and performs better at detecting the most advanced AAs, such as those with high-grade dysplasia (HGD) or size ≥2.0 cm (11, 12).

Cell-free DNA (cfDNA) consists of extracellular fragments of DNA present in blood and other bodily fluids. DNA fragments derived from apoptotic neoplastic cells (13) are commonly referred to as ctDNA. Classically, the presence of ctDNA fragments has been assessed through the analysis of somatic mutations, such as single-base substitutions, and via aneuploidy or gains and losses of chromosome arms, which are exquisitely specific markers of neoplasia. cfDNA fragmentation patterns have also been used to identify patients with cancer (14, 15). Such fragmentation patterns depend on several factors, including nucleosome organization, chromatin structure, gene expression, and nuclease content of the cell of origin of the cfDNA (14, 15). In addition, epigenetic alterations such as DNA methylation (16, 17), as well as elevation of some plasma proteins (18) have also been shown to be able to detect multiple different types of cancer.

Studies on blood-based screening have generally focused on detection of cancer. The few previous studies that investigated the detection of precursor lesions via a blood-based assay often reported low sensitivity. For example, only one of 11 patients with AAs had detectable ctDNA mutations in his/her plasma in a study performed in 2005 (19), whereas in a recent, large blood-based multiomic study of colorectal cancer involving 22,000 participants, the sensitivity for advanced pre-cancerous lesions was only 13%, barely higher than the fraction of control individuals that were positive (10%; ref. 20).

In the current study, we assessed the performance of four different methods to detect AAs in plasma: (i) a machine learning algorithm based on cfDNA fragmentation analysis, called *Signa*tures of fragment *L*ength (SignaL); (ii) circulating protein levels; (iii) aneuploidy scores; and (iv) cfDNA mutation analysis. Existing data from large study populations with and without cancer were utilized to determine 99.5% specificity thresholds.

## Materials and Methods

### AA study population

Study participants were patients evaluated with colonoscopy (all controls and 24/40 cases) or undergoing surgery for removal of a large AA (16/40 cases) at the University of Pittsburgh Medical Center facilities. None of the participants had a prior history of colorectal cancer. Cases were patients with an AA defined as an adenoma ≥10 mm in size or with tubulovillous or villous histology or with HGD. Controls were patients who had a negative colonoscopy (i.e., no adenomas or colorectal cancer detected during their colonoscopy). Blood samples were collected immediately prior to the procedures. All participants provided written informed consent and the institutional review board at the University of Pittsburgh approved the protocol. Laboratory personnel involved in the assessment of the specimens and the investigators that performed the data analysis were blinded to case–control status.

**Figure 1:**
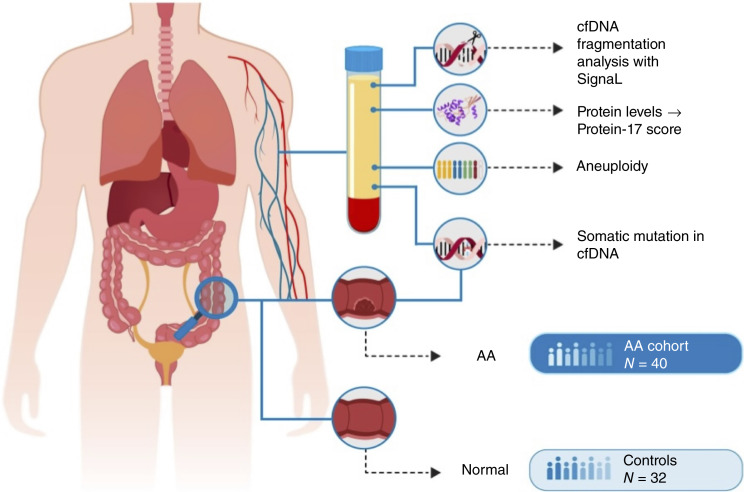
Summary of the overall investigative approach.

Figure 1 summarizes the overall investigative approach. Characteristics of the study population are described in Table 1.

**Table 1:** Adenoma characteristics of the study population, stratified by status

Characteristic	AA cases^a^ (*N* = 40)	Controls (*N* = 32)	*P* value^b^
Age (in years)			
Mean ± SD	63.8 ± 9.8	61.4 ± 10.2	0.32
Range	37–80	45–80	
Sex, *N* (%) female	23 (57.5)	16 (50.0)	0.53
Race, *N* (%)			0.73
African American	5 (12.5)	3 (9.4)	
White	35 (87.5)	29 (90.6)	
BMI (in kg/m^2^)			0.0005
Mean ± SD	32.1 ± 6.9	27.1 ± 4.4	
Range	20.8–49.8	19.6–36.8	
Smoking status, *N* (%)			0.33
Never	22 (55.0)	21 (65.6)	
Former	13 (32.5)	10 (31.3)	
Current	5 (12.5)	1 (3.1)	
Adenoma removal, *N* (%)		Not applicable	Not applicable
At colonoscopy	24 (60.0)		
At surgery	16 (40.0)		
≥1 AA present, *N* (% yes)	8 (20.0)	Not applicable	Not applicable
Adenoma size (cm)		Not applicable	Not applicable
1.0–1.5	10 (25.0)		
1.6–2.5	12 (30.0)		
2.6–3.5	9 (22.5)		
3.6–4.5	6 (15.0)		
≥4.6	3 (7.5)		
Histology, *N* (%)		Not applicable	Not applicable
Tubular	24 (60.0)		
Tubulovillous	9 (22.5)		
Villous	2 (5.0)		
Serrated	5 (12.5)		
Dysplasia, *N* (% high grade/severe)	8 (20.0)	Not applicable	Not applicable
Location, *N* (%)		Not applicable	Not applicable
Cecum	18 (45.0)		
Ascending colon	6 (15.0)		
Hepatic flexure	3 (7.5)		
Transverse colon	4 (10.0)		
Descending colon	1 (2.5)		
Sigmoid colon	2 (5.0)		
Rectum	6 (15.0)		

a If multiple AAs were present, information is provided for the most advanced AA (based on size, histology. and dysplasia).

b *T* test used for continuous variables; χ^2^ test or Fisher exact test for categorical variables.

### Blood processing

Plasma was collected in EDTA tubes prior to colonoscopy or surgery. Blood samples were processed immediately, and single-spun plasma was stored at −80°C until use. cfDNA was purified from 1 to 8 mL of plasma using either a QIASymphony Circulating DNA kit (Qiagen, cat #55114) or a BioChain Cell Free DNA Extraction kit (cat #K5011625) as specified by the manufacturers. cfDNA was quantified using qPCR using SsoAdvanced SYBR Green Supermix (Bio-Rad, cat #1725271) as directed by the manufacturer and employing the following primers:

1) 5′-CAC​ACA​GGA​AAC​AGC​TAT​GAC​CAT​GGG​TAA​CAG​CTT​TAT​CTA​TTG​ACA​TTA​TGC-3′
2) 5′-CGACGTAAAACGACGGCCAGTNNNNNNNNNNNNNNAAACTTCATGCTTCATCTAGTCAGC-3′.

### RealSeqS

Repetitive Element AneupLoidy Sequencing System (RealSeqS) was used to amplify ∼350,000 loci scattered throughout the genome, using a single primer pair, as previously described (21). PCR was performed in 25 μL reactions and amplification products were purified with AMPure XP beads (Beckman, cat #a63880). Massively parallel sequencing was performed on an Illumina HiSeq 4000 (21).

### SignaL fragmentation score

To assess cfDNA fragmentation patterns, we used RealSeqS (21) to amplify repeated elements in cfDNA from plasma. SignaL is a machine learning algorithm to identify signatures of fragment length in cfDNA and uses those signatures to classify samples as SignaL positive or negative. The input features of the SignaL algorithm are represented by an integer-valued vector in which every entry represents the read counts of the locus (amplicon) among the defined list of amplicons that RealSeq amplifies. We trained SignaL utilizing existing RealSeqS-generated sequencing data from 379 patients with stage I to III colorectal cancer and 327 individuals without known cancer to yield a fragmentation score. At a SignaL score of >0.78, corresponding to 99.5% specificity, 376 (97%) of the 379 patients with colorectal cancer and two (0.005%) of the controls scored positively (Supplementary Table S1).

Four steps were employed to generate the SignaL score:

1. Amplicon filtering. The purpose of filtering is to detect and eliminate amplicons that are sensitive to batch effects. Details of the filtering are presented in the Supplementary Data and illustrated in Supplementary Fig. S1.
2. Signature extraction. As illustrated in Supplementary Figs. S2 and S3 and detailed in the Supplementary Data, we extract signatures of fragmentation, defined as distributions over amplicons, from the amplicons read counts matrix. The choice of the number of dimensions/signatures is made using five-fold cross-validation on the training set. We choose the dimension such that the sensitivity obtained at 99.5% specificity plateaued. Details of the signature extraction are presented in the Supplementary Data and illustrated in Supplementary Figs. S2 and S3.
3. Feature extraction and selection. We select signatures consistent with the constraint that cancer samples have a more fragmented cfDNA. Details of the feature selection are presented in the Supplementary Data and illustrated in Supplementary Fig. S4.
4. Support vector machine (SVM). After selecting the features, we train an SVM (Supplementary Fig. S4) with a Gaussian kernel (R v3.4 e1071 library) to generate the SignaL score.

### Protein-17 score

Protein-17 is a machine learning algorithm that utilizes plasma levels of 17 cancer-associated proteins to generate a protein-specific score. The proteins targeted include myeloperoxidase, sex hormone–binding globulin, growth differentiation factor 15, neuron-specific enolase, osteoprotegerin, Dikkopf-1, α-fetoprotein, cancer antigen 125, cancer antigen 15-3, cancer antigen 19-9, carcinoembryonic antigen, hepatocyte growth factor, osteopontin, cytokeratin 19 fragment, interleukin-8, fibroblast growth factor 2, and tissue inhibitor of metalloproteinase 1. The protein-17 score was developed utilizing a training set comprising 388 patients with colorectal cancer and 812 individuals without cancer with existing plasma level data for the 17 proteins that have been previously described (18, 22). At a Protein-17 score of >0.96, corresponding to 99.5% specificity, 115 (29.6%) of the 388 patients with colorectal cancer and four (0.005%) of the 812 controls scored positively (Supplementary Table S2).

A subset of patients with colorectal cancer (*N* = 333) and individuals without cancer (*N* = 37) in the Protein-17 score training set had RealSeqS-generated data available as well and were also part of the SignaL training set. The methods for obtaining the Protein-17 score are described in the Supplementary Data.

### Aneuploidy

Based on the RealSeqS-generated sequencing data, a global aneuploidy score (GAS; refs. 21, 23), was generated for all patients in the AA study population. An SVM was (24) trained on existing RealSeqS data generated on 363 presumably euploid samples from individuals without cancer, 128 samples from patients with cancer, and 648 *in silico*–generated aneuploid samples derived from samples of individuals without cancer. The model was built using R v3.4 with the e1071 library and generated a GAS ranging from 0 to 1. From the training set, we established a GAS of >0.71, corresponding to 99.5% specificity, to define aneuploidy. The methods for obtaining a GAS are described in the Supplementary Data.

### Mutations in circulating cfDNA in plasma

For the SignaL, Protein-17, and aneuploidy assays, 1 mL of plasma was sufficient but optimal mutational analysis required a minimum of 8 mL (18, 22), which was available for 34/40 AA cases. In 2/34 AA cases, no mutations were detected in the AA tissue. Formalin-fixed, paraffin-embedded histologic sections of the AA tissue were macro-dissected under a microscope to ensure a neoplastic cellularity of >40%. DNA purified from the macro-dissected tissue was assessed for somatic mutations in 15 genes recurrently mutated in colorectal cancer: *SMAD4*, *TP53*, *AKT1*, *APC*, *BRAF*, *CTNNB1*, *ERBB3*, *FBXW7*, *HRAS*, *KRAS*, *NRAS*, *PIK3CA*, *PPP2R1A*, *RNF43*, and *POLE* (25–29).

The QIAamp Circulating Nucleic Acid Kit (Qiagen, cat #55114) was used to isolate cfDNA from plasma. To maximize sensitivity, we used a new variant of Safe Sequencing System (SafeSeqS) (30), called “multi-well SafeSeqS”, in which the cfDNA was distributed into 95 wells, and each well was independently amplified and sequenced. In each well, amplicons corresponding to the specific DNA mutations identified in the patient’s AA tissue were included, with up to 300 genome equivalents present in each well. This partitioning approach across a large number of wells raises the signal-to-noise ratio, resulting in a more sensitive mutation detection assay (31, 32).

Unlike the other assays, in which specificity can be evaluated in a population of subjects without cancer, it is impractical to test control individuals for each mutation and each combination of mutations identified within AA tissue. To establish that an identified mutation in plasma was likely to be a true mutation and not an artifact of sample preparation or sequencing, we produced primer-generated sequencing data from a control sample without cancer using the amplicons that were specific to the patient’s AA tissue mutations. We then performed a statistical analysis based on the mutant allele frequencies (MAF) obtained from each base pair within the entire amplicon primer-generated sequencing data. The MAF of each mutation was calculated and compared with the MAF of the control sample. Additionally, the MAFs of all other mutations that were observed within the PCR amplicons for the particular AA sample (i.e., those not corresponding to the mutations of interest) were calculated and used to model the assay-specific distribution of the assay’s background noise.

Two types of measurements were used to generate the distribution of the background assay noise:

1. The MAFs of mutations found in the patient’s plasma DNA that were not present in the corresponding adenomatous tissue and
2. The MAFs of all mutations (including the specific somatic mutations of interest identified by sequencing the adenomatous tissue) in the control sample.

We scored each mutation by comparing the MAF observed to the mutation’s background noise MAF distribution. We then computed an overall score aggregating the scores of all mutations identified in the AA tissue. We generate a *P* value associated with this score, in which the null distribution of the score assumes that all mutation MAFs are due to background noise. In other words, if any one mutation is real, we deviate from the null distribution. If the *P* value of the aggregate score is less than 0.01, the sample was designated as circulating-mutation positive. A summary of the multi-well SafeSeqs results, including the mutations analyzed for each patient and the call corresponding to the mutation for each well, and the final patient *P* values are included in Supplementary Table S3. A detailed description of the statistical model used for mutation analysis is provided in the Supplementary Data (Statistical Approach for Mutation Analysis).

This approach should be interpreted as an upper bound of the performance that can be achieved for circulating cfDNA mutations as a biomarker to detect AAs, and it is not applicable to a screening scenario because in screening prior knowledge of the mutations of interest is not possible. Moreover, restricting the set of mutations for every sample to the ones present in the AA tissue reduces the search horizon and dramatically reduces sources of noise.

### Statistical analyses

For analyses, AA location was consolidated into proximal colon (cecum, ascending, hepatic flexure, and transverse colon), and distal colon (descending, sigmoid, and rectum). Relationships between continuous variables and scores were evaluated using Pearson correlation tests; *t* tests were used for continuous variables and scores; and χ^2^ tests or Fisher exact tests were used for categorical variables. All proportion’s 95% confidence intervals (CI) were generated using Wilson score intervals. All *P* values were generated using the Scipy library (version 1.10.0) in Python (version 3.9.10). All other 95% confidence intervals were generated using the statsmodels library (version 0.14.0) in Python (version 3.9.10).

To minimize false positives, the primary analysis for each methodology used a specificity threshold of 99.5%, derived from the different training sets. In colorectal cancer screening, a positive biomarker test results in a recommendation for evaluation with colonoscopy. Colonoscopy is itself a recommended screening test for colorectal cancer, and many screening colonoscopy exams are negative (11). It is therefore reasonable to combine models using their respective thresholds and define a test as positive if at least one of the biomarkers is positive at the expense of specificity.

### Data availability

The data and code supporting this study are available in the following GitHub repository: https://github.com/klahoue1/Blood-based-Assay-for-Detection-of-Patients-with-Advanced-Adenomas.

## Results

The AA study population included 40 AA cases and 32 controls. Cases and controls were similar with respect to age, sex, race, and smoking status but had different body mass index (BMI; *P* = 5 × 10^−4^; Table 1). Importantly, BMI did not correlate with SignaL (see below). Sixteen (40.0%) cases had their AA removed during surgery; most AAs had tubular histology and most were smaller than 2.5 cm, and only eight (20%) showed high grade/severe dysplasia (Table 1). Adenoma characteristics including size, location, histopathologic type, and presence of dysplasia, and SignaL, Protein-17, and GAS scores are presented for each included case in Table 2.

**Table 2:** Adenoma characteristics and results of SignaL, Protein-17, and GAS (aneuploidy) scores. Full test set scores in Supplementary Tables S4 and S5

#	Age	Sex	Location	Dysplasia	Maximum adenoma size dimension (cm)	Histopathologic type	SignaL score	GAS score	Protein-17
1	59	Female	Rectum	High grade/severe	5.3	Tubular	0.96	0.75	0.17
2	63	Male	Sigmoid colon	Low	2.1	Tubulovillous	0.96	0.61	0.02
3	71	Male	Cecum	Low	4	Tubular	0.94	0.4	0.37
4	63	Male	Cecum	Low	1.6	Tubular	0.94	1	0.99
5	46	Female	Rectum	Low	4	Tubular	0.92	0.99	0.92
6	65	Male	Cecum	Low	2.5	Tubulovillous	0.92	0.98	1
7	74	Female	Cecum	Low	1.5	Tubular	0.85	0.99	0.8
8	52	Female	Rectum	Low	2.6	Tubular	0.82	0.83	0.78
9	66	Male	Cecum	Low	1.2	Tubular	0.79	0.78	0.43
10	56	Male	Ascending colon	Low	3.5	Tubular	0.63	0.29	0.04
11	65	Male	Hepatic flexure	Not available	2.5	Tubular	0.61	0.72	0.43
12	56	Male	Rectum	High grade/severe	6	Tubulovillous	0.6	0.92	0.3
13	60	Female	Transverse colon	Low	1.3	Tubular	0.52	0.54	0.25
14	64	Female	Transverse colon	Low	1	Tubular	0.47	0.07	0.52
15	52	Female	Ascending colon	High grade/severe	3.2	Tubular	0.45	0.39	0.96
16	72	Male	Cecum	High grade/severe	2	Tubular	0.41	0.15	0.27
17	67	Female	Cecum	Low	4.5	Tubulovillous	0.37	0.21	0.26
18	71	Female	Ascending colon	Low	3.2	Tubular	0.32	0.05	0.15
19	75	Male	Cecum	Low	1	Tubular	0.29	0.09	0.08
20	50	Female	Cecum	High grade/severe	4.5	Tubulovillous	0.26	0.67	0.99
21	61	Female	Rectum	Low	2.8	Villous adenoma	0.25	0.98	0.79
22	59	Female	Cecum	Low	1.2	Tubular	0.25	0.25	0.36
23	67	Male	Transverse colon	Low	1	Tubular	0.24	0.22	0.26
24	47	Female	Cecum	High grade/severe	5.2	Tubulovillous	0.22	0.29	0.22
25	50	Male	Sigmoid colon	Low	1.8	Tubular	0.18	0.37	0.04
26	53	Male	Descending colon	Low	2	Tubular	0.17	1	0.59
27	72	Female	Ascending colon	Not available	3	Tubular	0.16	0.42	0.36
28	80	Male	Cecum	High grade/severe	3	Tubular	0.15	0.44	0.99
29	71	Female	Cecum	Low	2	Serrated adenoma	0.15	0.35	0.08
30	37	Male	Cecum	Low	1.5	Serrated adenoma	0.12	0.2	0.11
31	78	Male	Transverse colon	Low	1.8	Tubulovillous	0.1	0.25	0.06
32	67	Female	Ascending colon	Low	3	Serrated adenoma	0.09	0.17	0.06
33	69	Male	Cecum	Low	4	Tubulovillous	0.09	0.19	0.87
34	76	Female	Cecum	Low	3	Tubular	0.07	0.15	0.12
35	68	Female	Rectum	Low	2.5	Villous adenoma	0.07	0.55	0.57
36	72	Female	Ascending colon	Low	2.5	Tubulovillous	0.05	0.58	0.93
37	65	Female	Hepatic flexure	Low	1.2	Serrated adenoma	0.03	0.18	0.21
38	68	Female	Cecum	High grade/severe	2.5	Tubular	0.02	0.26	0.27
39	74	Female	Cecum	Low	4	Tubular	0.01	0.05	0.92
40	72	Female	Hepatic flexure	Low	1.5	Serrated adenoma	0.01	0.13	0.13

### Relationship of patient and adenoma characteristics to assay scores

Age did not correlate with SignaL (*P* = 0.18), Protein-17 (*P* = 0.78), or GAS score (*P* = 0.08), and neither did adenoma size [SignaL (*P* = 0.42), Protein-17 (*P* = 0.40), and GAS (*P* = 0.41)] and BMI [SignaL (*P* = 0.32), Protein-17 (*P* = 0.13), and GAS (*P* = 0.33)]. No significant differences in SignaL, Protein-17, and GAS scores were observed between females and males (*P* = 0.12, *P* = 0.55, and *P* = 0.45, respectively) and between adenomas with HGD and adenomas with low-grade dysplasia (*P* = 0.97, *P* = 0.50, and *P* = 0.79, respectively). Serrated adenomas (*N* = 5) had significantly lower scores than tubular/tubulovillous adenomas (*N* = 35): SignaL (*P* = 0.09), Protein-17 (*P* = 0.03), and GAS (*P* = 0.02). All serrated adenomas were located in the proximal colon. After adjusting for serrated histology, there was no difference in SignaL (*P* = 0.21) and Protein-17 scores (*P* = 0.81) between proximal and distal colon locations. The GAS was significantly higher in the distal colon (*P* = 0.001).

### Detection of AAs by SignaL

**Figure 2:**
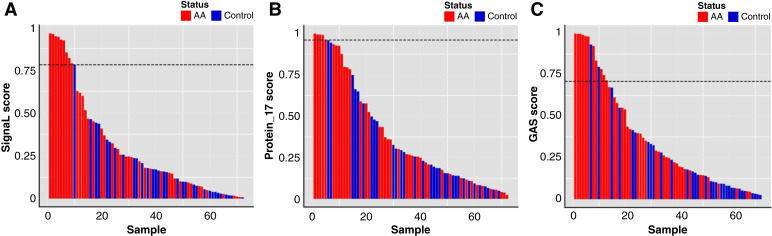
**A,** Sorted SignaL scores in plasma from 40 subjects with AAs (cases; in red) and 32 subjects with a normal colonoscopy (controls; in blue). The hatched line corresponds to a SignaL score >0.78, which detects 9/40 patients with AA (22.5%) at 100% specificity. **B,** Sorted Protein-17 scores in plasma samples from 40 subjects with AAs (cases; in red) and 32 subjects without adenomas (controls; in blue). The hatched line corresponds to a Protein-17 score >0.96, which detects 5/40 patients with AA (12.5%) at 100% specificity. **C,** Sorted GAS scores in plasma samples from 40 subjects with AAs (cases; in red) and 32 subjects without adenomas (controls; in blue). The hatched line corresponds to a GAS score >0.71, which detects 11/40 patients with AA (27.5%) at 93.8% specificity.

At a SignaL score of >0.78, corresponding to the 99.5% specificity threshold in the SignaL training set population without cancer, 9/40 patients with AA scored positively (22.5%, 95% CI, 12.3%–37.5%). None of the 32 individuals without adenomas scored positively (100% specificity, 95% CI, 89.3%–100%; Fig. 2A).

### Detection of AAs with Protein-17

At a Protein-17 score of >0.96, corresponding to the 99.5% specificity threshold in the Protein-17 training set population without cancer, 5/40 patients with AA scored positively (12.5%, 95% CI, 5.5%–26.6%; Fig. 2B; Supplementary Table S4). None of the 32 individuals without adenomas scored positively (100% specificity, 95% CI, 89.3%–100%). Of the five patients with AA detected with Protein-17, three were not identified by SignaL.

### Combination of SignaL with Protein-17

Using SignaL in combination with the Protein-17 assay at the 99.5% specificity threshold, 12/40 patients with AA (30%, 95% CI, 18.1%–45.4%) scored positively and 0/32 controls were positive.

### Detection of AAs with aneuploidy

At a GAS of >0.71, corresponding to 99.5% specificity in the GAS training set, 11/40 AA cases scored as aneuploid (27.5%, 95% CI, 16.1%–42.8%; Fig. 2C). However, in our sample at that GAS threshold (>0.71), 2/32 controls scored positively (specificity 93.8%, 95% CI, 79.9%–98.3%). Of the 11 samples scored as aneuploid, seven had a positive SignaL and two had a positive Protein-17 score at 99.5% specificity.

Using SignaL in combination with GAS, each at their respective 99.5% specificity thresholds, yielded 13/40 AA detected (32.5%, 95% CI, 20.1%–48%) with 2/32 false positives (specificity 93.8%, 95.5% CI, 79.9%–98.3%). In comparison with SignaL, at a similar specificity of 93.8% (2/32 positive controls), we detected 14/40 AAs (35%, 95% CI, 22.1%–50.5%).

### Combinations of SignaL, Protein-17, and aneuploidy

**Figure 3:**
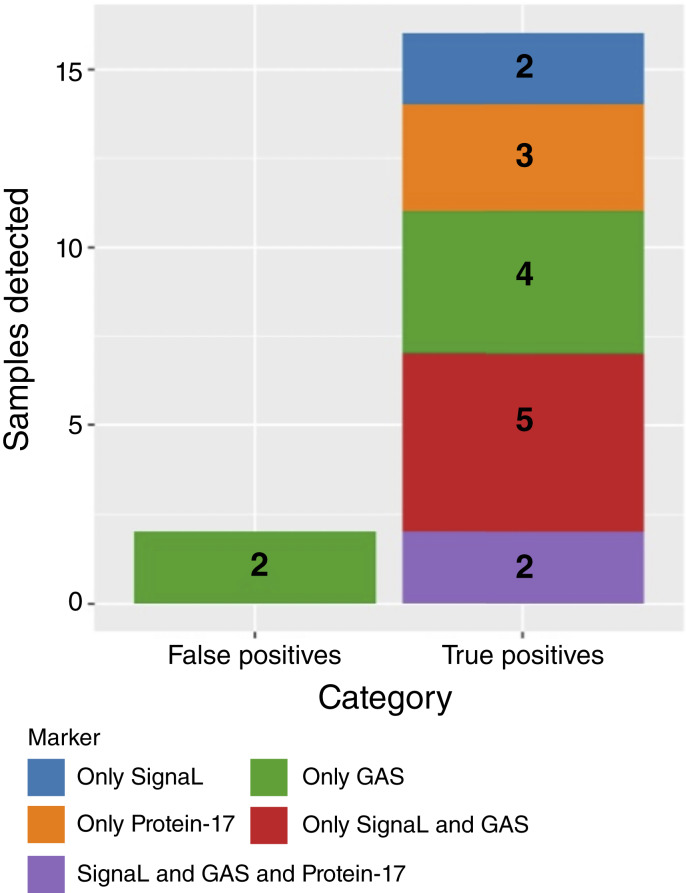
Cumulative barplot of the contributions of SignaL, Protein-17, and GAS to detection of AA at 99.5% specificity thresholds.

Table 3 details the performance of all biomarker combination at the 99.5% specificity threshold from the training sets. A combination of SignaL, GAS, and Protein-17 had the highest sensitivity (sensitivity 40%, 95% CI, 26.3–55.4) and detected 16/40 AAs, with two false positives from GAS, resulting in a specificity of 93.8% (95.5% CI, 79.9–98.3; Fig. 3). Table 3 also includes estimates for FIT and mt-sDNA performance in our study population.

**Table 3:** Performance of biomarker combinations at 99.5% specificity and comparison with FIT and mt-sDNA

	Sensitivity (%) and specificity (%)			
Methodology	All adenomas (*N* = 40)	HGD (*N* = 8)	≥2 cm (*N* = 19)	≥1 to <2 cm (*N* = 13)
SignaL	22.5, 100	12.5, 100	26.3, 100	23, 100
Protein-17	12.5, 100	37.5, 100	5.3, 100	7.6, 100
GAS	27.5, 93.8	25, 93.8	31.6, 93.8	23, 93.8
SignaL + Protein-17	30, 100	50, 100	26.3, 100	23, 100
SignaL + GAS	32.5, 93.8	25, 93.8	42.1, 93.8	23, 93.8
Protein-17 + GAS	35, 93.8	62.5, 93.8	26.3, 100	23, 93.8
SignaL + Protein-17 + GAS	40, 93.8	62.5, 93.8	42.1, 93.8	23, 93.8
FIT^a^	37.5, 96	50, 96	42.1, 96	23.1, 96
Cologuard (mt-sDNA)^a^	60, 90	75, 90	68.4, 90	38.5, 90
Cologuard Next-generation^b^	60, 92.7	75, 92.7	68.4, 92.7	38.5, 92.7

a Performance is estimated based on sensitivity and specificity within adenoma subtypes by Imperiale and colleagues (11).

b Performance based on Imperiale and colleagues (12).

### Detection of AAs by cfDNA mutations in plasma

Of the 15 genes commonly mutated in colorectal cancer that were evaluated, at least one mutation was identified in 32/34 (94.1%) of the available AA tissues. The average number of mutations detected was 2.53 per adenoma and ranged from one to seven for a total of 53 mutations in nine different genes (Supplementary Table S3). Despite using the more sensitive multi-well SafeSeqS approach, and despite using primers for mutations that were already identified and known to be present in the AA tissue and hence potentially circulating as cfDNA, only one was cfDNA mutation positive at a level of significance that is 0.01 (Supplemenatry Table S3).

### Reproducibility of SignaL

**Figure 4:**
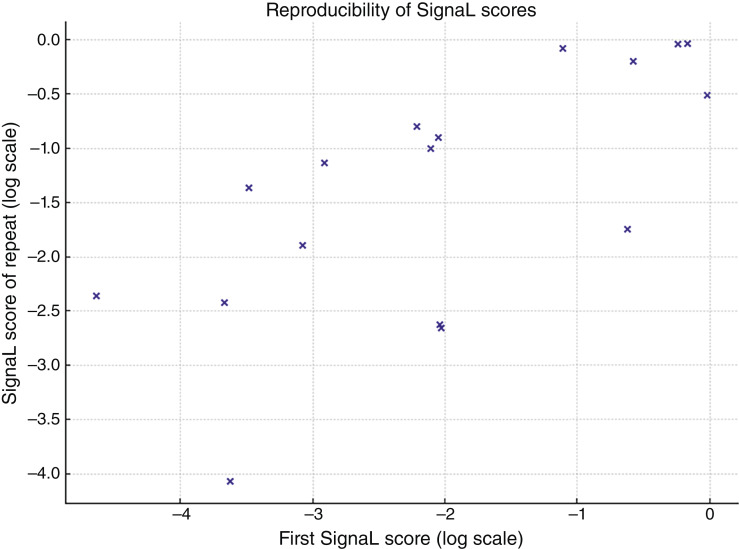
Comparison of SignaL scores in repeated plasma samples from 17 subjects with AAs. The scores are plotted in log scale.

Duplicate plasma aliquots from 17 case subjects were evaluated to test the reproducibility of SignaL. The DNA purification, RealSeqS amplification, and sequencing of these duplicate samples were done independently at a different time point. There was a significant correlation (*r* = 0.73; *P* = 0.0008) between the two independently obtained SignaL scores (Fig. 4). Replacing the 17 case subject scores by their duplicate plasma aliquot scores yielded 9/40 patients with AA scoring positively (22.5%, 95% CI, 12.3%–37.5%) when using the 99.5% specificity threshold. Seven of the nine case subjects scored positively in both plasma aliquots. Six of the eight case subjects scored negatively in both plasma aliquots.

## Discussion

In this case–control study, we assessed four different methods for AA detection in plasma. With SignaL, we achieved a 22.5% sensitivity for detection of AAs at 100% specificity. Combined with the Protein-17 assay, the sensitivity increased to 30.0% while maintaining 100% specificity. Combining SignaL, Protein-17, and GAS yielded a sensitivity of 40% at a 93.8% specificity.

A contextual benchmark for our test can be given by the performance of stool-based testing with FIT or mt-sDNA in detection of AA. Given that we did not have corresponding stool samples, we could not directly evaluate FIT and mt-sDNA in our study cohort and, therefore, used available information to estimate their performance. The sensitivity of mt-sDNA (Cologuard) for AAs with HGD ≥2.0 cm or 1.0 to 2.0 cm is 69%, 68%, and 38%, respectively, at 90% specificity with a normal colonoscopy exam (11). The corresponding results for FIT are 46%, 42%, and 21% at 96% specificity (11). Applying these sensitivities adjusted to the characteristics of our AA population estimates that mt-sDNA would detect 24/40 (60%) and FIT would detect 15/40 (37.5%) of our AAs at 96% specificity (Table 3). A next-generation mt-sDNA test, Cologuard Next Generation, reported a higher sensitivity for detection of AAs with HGD at 74.6% but similar sensitivity for AAs ≥2.0 cm (68%) or those between 1.0 to 2.0 cm (38%) at a higher specificity of 92.7% (12). After accounting for our enriched population of more advanced AAs such as those ≥2.0 cm or with HGD, our combined sensitivity of 40% is between the expected FIT (37.5%) and mt-sDNA (60%) sensitivity, at a specificity of 93.8%, which is between the specificity of mt-sDNA (90%) and FIT (96%). It is important to note that the stool-based studies were prospective, whereas ours is a case–control study. Therefore, the performance of our blood-based assays relative to stool testing is only suggestive. Large, prospective studies are required to determine whether our approach can add to the options currently available for screening patients for premalignant lesions of the colon.

We intentionally included many larger AAs (≥2.0 cm) and those with HGD in our study population because evidence suggests these characteristics are more strongly associated with risk of colorectal cancer (33). In addition, we examined our results at an expected 99.5% specificity threshold to examine test performance for inclusion within a multicancer early detection assay (18). In that context, one would want to maintain a high specificity to minimize false-positive findings.

Identification and removal of adenomas have a major impact on the benefit of screening in reducing colorectal cancer mortality (4, 5). Modeling studies demonstrate that primary prevention via the detection of advanced precancerous lesions by blood-based assays would significantly enhance their effectiveness in reducing colorectal cancer incidence and mortality (34, 35) and is essential to their cost effectiveness relative to current stool- and endoscopy-based screening tests (35, 36). In an initial report of a blood-based assay for colorectal cancer in more than 22,000 individuals, the sensitivity for advanced precancerous lesions was only 13%, and given the overall specificity of 90%, indicates relatively little discrimination for AAs (20). Higher sensitivity for a blood-based detection of AAs is a priority for effective blood-based screening.

Consistent with previous studies (19), and despite the use of a highly sensitive partitioning assay focused on known mutations present in AA tissue, circulating cfDNA mutations were reliably identifiable in only a single patient of 32 tested at 0.01 level of significance. Limiting the search to mutations known to be present in AA tissue is not a feasible strategy for screening and as such, we did not include cfDNA mutations in our evaluation of combinations of testing methods. The cfDNA analysis was used to maximally estimate the potential yield of mutation testing. It was clearly less sensitive than the other assays examined.

A particular strength of this study was the use of independent populations of hundreds of subjects without cancer to establish the thresholds for positivity for the assays under study. These thresholds were unrelated to our AA test set. Other strengths include novel methodologic developments in assessing cfDNA fragmentation and a highly sensitive approach for identifying circulating mutations in plasma.

There are also several limitations. Larger, prospective studies are needed to confirm our results, which were based on evaluation of only 72 subjects. Testing in subjects with multiple non-AAs and in larger numbers of subjects with serrated adenomas is needed. All our algorithms were trained on plasma samples from patients with cancer and subjects without cancer. It will be important to include controls with various comorbidities in future prospective studies, so as to confirm the specificity in typical screening populations of older individuals. Conversely, it is conceivable that a higher sensitivity of SignaL, Protein-17, and/or GAS could be obtained by training on samples from patients with AAs as opposed to those with cancer. To support this statement, we conducted a preliminary exploratory analysis, which suggests that including patients with AAs would improve sensitivity (Supplementary Fig. S5).

A potential limitation of SignaL is that the cell of origin responsible for positivity is unknown. As with all fragmentation-based tests of cfDNA, the positivity is likely related to abnormal chromatin or nucleases in the cells giving rise to the positive signal (37). Because a positive result with SignaL was found more often than a positive result with assays of genetic abnormalities (i.e., aneuploidy or mutations), it is possible that the cell of origin is not neoplastic. We did not observe a significant association between adenoma size or histologic advancement, such as the degree of dysplasia, and the SignaL score. One can speculate that the source is inflammatory or immune cells within the tumor microenvironment or even a systemic response initiated by the AA neoplastic growth. These are fertile areas for future research (37, 38).

The enrichment of our test set with very large adenomas with HGD was purposeful as we assumed that if we could not detect larger, more advanced AAs, which are closer to malignant transformation, the approach would not be worthy of further pursuit in the population of AAs more typically encountered during screening, which include adenomas 1 to 1.5 cm in size. However, we generally did not observe associations between adenoma characteristics and our assay scores.

In conclusion, a subset of AAs can specifically be detected via analysis of plasma cfDNA fragmentation, cancer-associated proteins, and aneuploidy. Further evaluation of this approach could improve blood-based testing for colorectal cancer.

## Supplementary Material

Supplementary Data Supplementary Appendix

Figure S1 Supplementary Figure 1: Illustration of the RealSeqS amplicons filtering process.

Figure S2 Supplementary Figure S2: Illustration of the signatures extraction.

Figure S3 Supplementary Figure S3: Non-negative factorization of the counts matrices. Every chromosomal arm is represented by a matrix. Before factorization, the amplicon counts are normalized via dividing the raw counts by the total number of counts coming from each chromosomal arm.

Figure S4 Supplementary Figure S4: Illustration of the SignaL features extraction and selection procedure.

Figure S5 Supplementary Figure S5: Sorted SignaL scores in plasma from 20 subjects with advanced adenomas, 20 subjects with colorectal cancer, and 32 control subjects, after including 20 subjects with advanced adenoma in the training set.

Supplemental Table 1 SignaL scores for every sample in the SignaL training set (N=706).

Supplemental Table 2 Protein levels and Protein-17 score for every sample in the protein training set (N=1200).

Supplemental Table 3 Mutations detected, mutation calls at individual well level, and overall mutation score for every tested sample (N=32).

Supplemental Table 4 Protein levels and Protein-17 score for every sample in the proteins test set (N=72).

supplemental table 5 SignaL and GAS scores of the test set (N=72)

Supplementary Data Statistical Approach for Mutations

## References

[1] Siegel RL , Miller KD, Wagle NS, Jemal A. Cancer statistics, 2023. CA Cancer J Clin 2023;73:17–48. 36633525 10.3322/caac.21763

[2] Atkin WS , Edwards R, Kralj-Hans I, Wooldrage K, Hart AR, Northover JMA, . Once-only flexible sigmoidoscopy screening in prevention of colorectal cancer: a multicentre randomised controlled trial. Lancet 2010;375:1624–33. 20430429 10.1016/S0140-6736(10)60551-X

[3] Schoen RE , Pinsky PF, Weissfeld JL, Yokochi LA, Church T, Laiyemo AO, . Colorectal-cancer incidence and mortality with screening flexible sigmoidoscopy. N Engl J Med 2012;366:2345–57. 22612596 10.1056/NEJMoa1114635 PMC3641846

[4] Segnan N , Armaroli P. Early detection versus prevention in colorectal cancer screening: methods estimates and public health implications. Cancer 2017;123:4767–9. 28976554 10.1002/cncr.31032

[5] Doroudi M , Schoen RE, Pinsky PF. Early detection versus primary prevention in the PLCO flexible sigmoidoscopy screening trial: which has the greatest impact on mortality? Cancer 2017;123:4815–22. 28976536 10.1002/cncr.31034 PMC5716922

[6] Ladabaum U , Dominitz JA, Kahi C, Schoen RE. Strategies for colorectal cancer screening. Gastroenterology 2020;158:418–32. 31394083 10.1053/j.gastro.2019.06.043

[7] Joseph DA , King JB, Dowling NF, Thomas CC, Richardson LC. Vital signs: colorectal cancer screening test use—United States, 2018. MMWR Morb Mortal Wkly Rep 2020;69:253–9. 32163384 10.15585/mmwr.mm6910a1 PMC7075255

[8] He X , Hang D, Wu K, Nayor J, Drew DA, Giovannucci EL, . Long-term risk of colorectal cancer after removal of conventional adenomas and serrated polyps. Gastroenterology 2020;158:852–61. 31302144 10.1053/j.gastro.2019.06.039 PMC6954345

[9] Click B , Pinsky PF, Hickey T, Doroudi M, Schoen RE. Association of colonoscopy adenoma findings with long-term colorectal cancer incidence. JAMA 2018;319:2021–31. 29800214 10.1001/jama.2018.5809 PMC6583246

[10] Lee JK , Jensen CD, Levin TR, Doubeni CA, Zauber AG, Chubak J, . Long-term risk of colorectal cancer and related death after adenoma removal in a large, community-based population. Gastroenterology 2020;158:884–94. 31589872 10.1053/j.gastro.2019.09.039 PMC7083250

[11] Imperiale TF , Ransohoff DF, Itzkowitz SH, Levin TR, Lavin P, Lidgard GP, . Multitarget stool DNA testing for colorectal-cancer screening. N Engl J Med 2014;370:1287–97. 24645800 10.1056/NEJMoa1311194

[12] Imperiale TF , Porter K, Zella J, Gagrat ZD, Olson MC, Statz S, . Next-generation multitarget stool DNA test for colorectal cancer screening. N Engl J Med 2024;390:984–93. 38477986 10.1056/NEJMoa2310336

[13] Cohen JD , Diergaarde B, Papadopoulos N, Kinzler KW, Schoen RE. Tumor DNA as a cancer biomarker through the lens of colorectal neoplasia. Cancer Epidemiol Biomarkers Prev 2020;29:2441–53. 33033144 10.1158/1055-9965.EPI-20-0549 PMC7710619

[14] Ivanov M , Baranova A, Butler T, Spellman P, Mileyko V. Non-random fragmentation patterns in circulating cell-free DNA reflect epigenetic regulation. BMC Genomics 2015;16:S1. 10.1186/1471-2164-16-S13-S1 PMC4686799 26693644

[15] Snyder MW , Kircher M, Hill AJ, Daza RM, Shendure J. Cell-free DNA comprises an in vivo nucleosome footprint that informs its tissues-of-origin. Cell 2016;164:57–68. 26771485 10.1016/j.cell.2015.11.050 PMC4715266

[16] Liu MC , Oxnard GR, Klein EA, Swanton C, Seiden MV; CCGA Consortium. Sensitive and specific multi-cancer detection and localization using methylation signatures in cell-free DNA. Ann Oncol 2020;31:745–59. 33506766 10.1016/j.annonc.2020.02.011 PMC8274402

[17] Okugawa Y , Grady WM, Goel A. Epigenetic alterations in colorectal cancer: emerging biomarkers. Gastroenterology 2015;149:1204–25. 26216839 10.1053/j.gastro.2015.07.011 PMC4589488

[18] Cohen JD , Li L, Wang Y, Thoburn C, Afsari B, Danilova L, . Detection and localization of surgically resectable cancers with a multi-analyte blood test. Science 2018;359:926–30. 29348365 10.1126/science.aar3247 PMC6080308

[19] Diehl F , Li M, Dressman D, He Y, Shen D, Szabo S, . Detection and quantification of mutations in the plasma of patients with colorectal tumors. Proc Natl Acad Sci U S A 2005;102:16368–73. 16258065 10.1073/pnas.0507904102 PMC1283450

[20] Chung DC , Gray DM, Singh H, Issaka RB, Raymond VM, Eagle C, . A cell-free DNA blood-based test for colorectal cancer screening. N Engl J Med 2024;390:973–83. 38477985 10.1056/NEJMoa2304714

[21] Douville C , Cohen JD, Ptak J, Popoli M, Schaefer J, Silliman N, . Assessing aneuploidy with repetitive element sequencing. Proc Natl Acad Sci U S A 2020;117:4858–63. 32075918 10.1073/pnas.1910041117 PMC7060727

[22] Lennon AM , Buchanan AH, Kinde I, Warren A, Honushefsky A, Cohain AT, . Feasibility of blood testing combined with PET-CT to screen for cancer and guide intervention. Science 2020;369:eabb9601. 32345712 10.1126/science.abb9601 PMC7509949

[23] Douville C , Springer S, Kinde I, Cohen JD, Hruban RH, Lennon AM, . Detection of aneuploidy in patients with cancer through amplification of long interspersed nucleotide elements (LINEs). Proc Natl Acad Sci U S A 2018;115:1871–6. 29432176 10.1073/pnas.1717846115 PMC5828610

[24] Mohri M , Rostamizadeh A, Talwalkar A. Foundations of machine learning. Cambridge (MA): MIT Press; 2018.

[25] Tie J , Cohen JD, Lahouel K, Lo SN, Wang Y, Kosmider S, . Circulating tumor DNA analysis guiding adjuvant therapy in stage II colon cancer. N Engl J Med 2022;386:2261–72. 35657320 10.1056/NEJMoa2200075 PMC9701133

[26] Diaz LA Jr , Williams RT, Wu J, Kinde I, Hecht JR, Berlin J, . The molecular evolution of acquired resistance to targeted EGFR blockade in colorectal cancers. Nature 2012;486:537–40. 22722843 10.1038/nature11219 PMC3436069

[27] Bettegowda C , Sausen M, Leary RJ, Kinde I, Wang Y, Agrawal N, . Detection of circulating tumor DNA in early- and late-stage human malignancies. Sci Transl Med 2014;6:224ra24. 10.1126/scitranslmed.3007094 PMC4017867 24553385

[28] Kinde I , Bettegowda C, Wang Y, Wu J, Agrawal N, Shih IM, . Evaluation of DNA from the papanicolaou test to detect ovarian and endometrial cancers. Sci Transl Med 2013;5:167ra4. 10.1126/scitranslmed.3004952 PMC3757513 23303603

[29] Tie J , Wang Y, Tomasetti C, Li L, Springer S, Kinde I, . Circulating tumor DNA analysis detects minimal residual disease and predicts recurrence in patients with stage II colon cancer. Sci Transl Med 2016;8:346ra92. 10.1126/scitranslmed.aaf6219 PMC5346159 27384348

[30] Kinde I , Wu J, Papadopoulos N, Kinzler KW, Vogelstein B. Detection and quantification of rare mutations with massively parallel sequencing. Proc Natl Acad Sci U S A 2011;108:9530–5. 21586637 10.1073/pnas.1105422108 PMC3111315

[31] Vogelstein B , Kinzler KW. Digital PCR. Proc Natl Acad Sci U S A 1999;96:9236–41. 10430926 10.1073/pnas.96.16.9236 PMC17763

[32] Morley AA . Digital PCR: a brief history. Biomol Detect Quantif 2014;1:1–2. 27920991 10.1016/j.bdq.2014.06.001 PMC5129430

[33] Wieszczy P , Kaminski MF, Franczyk R, Loberg M, Kobiela J, Rupinska M, . Colorectal cancer incidence and mortality after removal of adenomas during screening colonoscopies. Gastroenterology 2020;158:875–83. 31563625 10.1053/j.gastro.2019.09.011

[34] Ladabaum U , Church TR, Feng Z, Ransohoff, DF, Schoen, RE. Counting advanced precancerous lesions as true positives when determining colorectal cancer screening test specificity, J Natl Cancer Inst 2022;114:1040–3. 35134969 10.1093/jnci/djac027 PMC9275773

[35] Aziz Z , Wagner S, Agyekum A, Pumpalova YS, Prest M, Lim F, . Cost-effectiveness of liquid biopsy for colorectal cancer screening in patients who are unscreened. JAMA Netw Open 2023;6:e2343392. 37971743 10.1001/jamanetworkopen.2023.43392 PMC10654798

[36] Ladabaum UMA , Mannalithara A, Weng Y, Schoen RE, Dominitz JA, Desai M, . Comparative effectiveness and cost-effectiveness of colorectal cancer screening with blood-based biomarkers (liquid biopsy) vs. fecal tests or colonoscopy. Gastroenterology 2024;167:378–91. 38552670 10.1053/j.gastro.2024.03.011 PMC12279009

[37] Lo YMD , Han DSC, Jiang P, Chiu RWK. Epigenetics, fragmentomics, and topology of cell-free DNA in liquid biopsies. Science 2021;372:eaaw3616. 33833097 10.1126/science.aaw3616

[38] Fox-Fisher I , Shemer R, Dor Y. Epigenetic liquid biopsies: a novel putative biomarker in immunology and inflammation. Trends Immunol 2023;44:356–64. 37012121 10.1016/j.it.2023.03.005

