# Supplementary material for: A Blood-Based Assay for Detection of Patients with Advanced Adenomas
Source: Cancer Res Commun. 2025 Apr 16;5(4):621–31. doi: 10.1158/2767-9764.CRC-24-0398 (PMC12001750; doi:10.1158/2767-9764.CRC-24-0398)
Supplement: Supplementary Data — Supplementary Appendix [file crc-24-0398_supplementary_data_suppsd1.docx]

Supplement to Lahouel K, et al. **Detection of Advanced Adenoma in Plasma**

**Contents:**

Methods Supplement: SignaL Pages 3-4

Methods Supplement: Protein 17 Pages 4-5

Methods supplement: Aneuploidy Pages 5-6

Methods supplement: Circulating ctDNA mutations in plasma Page 6

Methods supplement: Advanced adenomas in the training set of SignaL Pages 6-7

**Supplementary Figures:**

**Supplementary Figure S1**: Illustration of the RealSeqS amplicons filtering process.

**Supplementary Figure S2:** Illustration of the signatures extraction.

**Supplementary Figure S3:** Non-negative factorization of the counts matrices. Every chromosomal arm is represented by a matrix. Before factorization, the amplicon counts are normalized via dividing the raw counts by the total number of counts coming from each chromosomal arm.

**Supplementary Figure S4:** Illustration of the SignaL features extraction and selection procedure.

**Supplementary Figure S5:** Sorted SignaL scores in plasma from 20 subjects with advanced adenomas, 20 subjects with colorectal cancer, and 32 control subjects, after including 20 subjects with advanced adenoma in the training set.

**Supplemental Tables:**

**Supplemental Table 1**: SignaL Training Set Scores

Description: SignaL scores for every sample in the SignaL training set (N=706).

**Supplemental Table 2**: Training Set Protein Levels

Description: Protein levels and Protein-17 score for every sample in the protein training set (N=1200).

**Supplemental Table 3**: Mutation Calls and Scores

Description: Mutations detected, mutation calls at individual well level, and overall mutation score for every tested sample (N=32).

**Supplemental Table 4**: Test Set Protein Levels

Description: Protein levels and Protein-17 score for every sample in the proteins test set (N=72).

**Supplemental Table 5**: SignaL and GAS scores of the test set (N=72).

1. **SignaL**

**Amplicon Filtering**

As illustrated in the flowchart (Supplementary Figure S1), we begin by splitting the data, i.e. the amplicon counts for every patient, by chromosome. We use the normal samples of the training set during the filtering step. To detect amplicons that are affected by batch effects, we use 3 different criteria for every chromosome. The first criterion aims at detecting different kinds of batch effects coming from known or unknown factors. The 2 other criteria eliminate respectively batch effects related to the sample ancestry and the primer lot used during PCR. More precisely, for the first filter, we compute for every amplicon the correlation between the counts of the amplicon and the total counts of the corresponding chromosome. If there is a strong correlation, this indicates that the main factor of variation of the amplicon counts among normal samples is the total number of counts. Normalizing the counts via dividing by the total counts of the chromosome will remove a major part of batch effects for those amplicons. Therefore, the first filter eliminates all amplicons with correlation less than a threshold $\rho_{0}$ that is taken to be 0.8 in our algorithm. The second filter targets specifically ethnicity as a factor of batch effects. For that, we run an ANOVA for every amplicon where the response variable is the normalized count of the amplicon, and the corresponding factor is the race of the patient. We only keep patients mapped to ethnicities represented with more than 10 samples among the normal samples of the training set. After running the ANOVA, the second filter eliminates the amplicons with the 20% lowest p-values. Finally, the third and last filter is identical to the second one with the difference that the considered factor is the primer lot used.

**Signatures Extraction**

As illustrated in the flowchart (Supplementary Figure S2), we begin by splitting the amplicon counts by chromosomal arms. We will represent the amplicon counts data by a matrix for every chromosome arm. To fix ideas, assume that every column represents a sample (here from the training set) and every row an amplicon (from the filtered amplicons). If we denote by $C$ the matrix of counts for a particular chromosome arm, we obtain the signatures by performing a non-negative matrix factorization (NMF) of the matrix $C \approx W H$ (Supplementary Figure S3). For this task, we used the R package “NMF”. The columns of the matrix $W$ are defined as the signatures of length of the corresponding chromosomal arm. The choice of the number of dimensions/signatures is made using 5-fold cross-validation. We choose the dimension such that the sensitivity obtained at 99% specificity plateaus. At this point, it is important to mention that we will need an additional iteration aiming at using the matrix $H$ of intensities as the features matrix. This part is described in the next section.

**Features extraction and selection**.

In this step, we use the signatures obtained from the previous step to obtain the features via an extra iteration of the non-negative factorization of the counts matrix. More precisely, for every counts matrix $C$ (for every chromosome arm from the training and test set), we find the matrix $H$ such that H minimizes:

$$\left| \left| C-WH \right| \right|^{2}$$

Under the constraints all the entries of $H$ are non-negative. This problem is the non-negative least squares problem (NNLS). Notice that the matrix $W$ is fixed in this problem. Every column of this matrix is a signature obtained from the previous step. We solve the NNLS using the R package “nnls”.

Solving the NNLS for every chromosomal arm and for both training and test sets therefore generates the features of SignaL. We finish this step by a features selection procedure. To fix ideas, we recall that one signature is one column with positive entries, where each entry corresponds to an amplicon. When we divide by the sum of the entries of the column, we obtain a probability distribution over amplicons. Since every amplicon has a defined length, a signature implies a distribution over lengths (of amplicons). As a consequence, we can define a mean length for every signature. For every chromosome arm, we partition the signatures into 3 types: the short signatures are signatures having an associated length in the bottom third of signature lengths, the long signatures having an associated length in the top third of signature lengths and finally the neutral signatures that are neither short neither long. Fragmentation among cancer samples is more intense than fragmentation of DNA among normal samples. For that, we impose that the features associated to short signatures have higher values among cancer samples, and the features associated to long signatures have higher values among normal samples. To compare values of features, we use the median value for every feature among normal samples and among cancer samples. All the features that do not satisfy this constraint are then excluded. Notice that all features associated to neutral signatures are kept in this procedure.

**Support Vector Machine (SVM)**

After selecting the features, we train an SVM (*1*) (Supplementary Figure S4) with a Gaussian kernel (R v3.4 e1071 library) to generate the SignaL score.

1. **Protein-17 Score**

We obtain the following 12 proteins with non-zero coefficients (i.e used in the score) in increasing order of coefficient magnitude: IL-8, GDF-15, CEA, MPO, CA-125, CA19-9, FGF2, TIMP-1, CYFRA21-1, SHBG, HGF, and OPN.

The classifier that we train to generate a proteins score is an elastic net (*2*) classifier with constraints on the coefficients. More precisely, let us denote by:

$$l\left( X,\beta\right)=\frac{exp\{\beta_{0}+\sum_{i=1}^{n} X_{i}\beta_{i}\}}{1+exp\{\beta_{0}+\sum_{i=1}^{n} X_{i}\beta_{i}\}}$$

Where $X$ represents the proteins/features vector, $X_{i}$ represents the ith coordinate of $X$ and $\beta$ is a coefficients vector. The probability of cancer/advanced adenomas given $X$ is equal to $l\left( X,\beta\right)$ and is also the score associated to proteins. To estimate $\beta$, we use the R package “glmnet” to solve the optimization problem over $\beta$ of the objective function:

$$\sum_{i=1}^{n} Y^{i}log(l(X^{i},\beta)+\sum_{i=1}^{n} (1-Y^{i})(1-log(l(X^{i},\beta))+\lambda(\left| \left| \beta\right| \right|_{1}+\frac{\left| \left| \beta\right| \right|_{2}^{2}}{2})$$

where $X^{i}$ is the ith sample of the training set and $Y^{i}$ is the corresponding label. We impose the condition that the coordinates of $\beta$ are positive. This corresponds to the prior idea that protein levels should be higher in advanced adenomas and cancer samples. Protein-17 Scores of the 72 patients of the test set are in (supp. table 4) of the main text.

1. **Aneuploidy as defined by RealSeqS**

An algorithm to detect the presence of aneuploidy from amplicon sequencing data has been described (*3, 4*). Our approach uses the normalized read counts of 500-kb intervals across the genome and performs a “within-sample” comparison. Outlier intervals are bioinformatically identified and filtered. Any aberrant segment <5 MB is assumed to be a germline copy number variation and bioinformatically removed. The statistical significance is then calculated for the chromosome arm without the aberrant <5 MB segments. The 39 non-acrocentric chromosome arm statistical significances are then used as predictive features in a supervised machine learning model. A support vector machine (SVM) (*1*) was trained on 363 presumably euploid samples from individuals without cancer, from 128 samples of cancer patients, and from 648 in silico-generated aneuploid samples derived from the samples of individuals without cancer. The model was built using R v3.4 with the e1071 library and generates a Global Aneuploidy Score (GAS) which ranges from 0 to 1. Several studies have evaluated copy number alterations in clonal hematopoiesis of indeterminate potential (CHIP) in large population databases (*5*) (*6, 7*). We initially did not explicitly account for the possibility of CHIP but many of the candidate regions are <5MB and would already be excluded using our previously described bioinformatic filters. Based on the new CHIP studies, we instituted one additional bioinformatic filter to limit the possibility that a positive GAS was derived from CHIP. CHIP samples most frequently exhibit only one aneuploid arm and is limited to a small number of candidate arms (*8*). Any aneuploid sample (positive GAS) with only one aneuploid arm from chr 5q, 13q, 14q, 20q was therefore bioinformatically labeled as CHIP and the GAS set to 0. GAS scores of the training set patients are in (supp. table 1). GAS Scores of the 72 patients of the test set are in (supp. table 5) of the main text.

1. **Assessment of** **circulating ctDNA mutations in plasma**

For each mutation we estimated a null distribution that is a mixture of the event of observing a zero MAF and a positive MAF modeled by a mixture of gaussians fit to the non-zero MAF observations. The parameters of this null distribution were estimated via the control sample compared to the AA sample and are specific to the particular mutation that is analyzed to account for the heterogeneity of PCR error rate across mutations. MAF thresholds yielding a p-value of 0.05 were determined from these mutation-specific distributions, and intermediate scores were calculated as the negative of the logarithm of the observed MAF p-value if the MAF is greater than the corresponding threshold and zero otherwise. To account for the number of genomic equivalents in each well, a second (additional) MAF threshold was determined from a second null distribution estimated using “spike-in” controls (details in Statistical Approach for Mutation Analysis). Using this distribution, we calculated an MAF threshold corresponding to a probability of 0.005 for observing an MAF lower than the experimentally determined value, conditioned on the initial number of genomic equivalents containing a single mutant template molecule. That is, if one mutant template molecule was present in a well containing the given number of genomic equivalents, the probability of observing an MAF below the determined threshold value is equal to 0.005. Finally, the score for each mutation in each well, is equal to the intermediate score described above if the MAF is higher than the second threshold, and zero otherwise.

Second, a cumulative score for each mutation was then calculated by summing the 95 values determined for each well. To estimate the null distribution of cumulative mutation scores, we applied this procedure to all mutations that were found in the patient’s plasma but were not identified in the corresponding tumor tissue. A p-value for each mutation of interest was then calculated from this empirical distribution.

Finally, the p-values corresponding to the mutations of interest were combined using Fisher’s method (*9*) to calculate a final p-value for the sample.

1. **Advanced adenomas in the training set of SignaL**

One limitation of our study is the absence of subjects with advanced adenoma in the training set for the SignaL algorithm. Including a subset of the 40 subjects with advanced adenomas in the training set would compromise our constraint of maintaining an independent validation cohort. However, we conducted an exploratory analysis, reported here but excluded from the main text, to assess the impact of incorporating advanced adenomas into the training set. Specifically, we randomly selected 50% of the 32 controls and 50% of the 40 advanced adenomas from the validation set (matched for gender and high-grade dysplasia status) and included them in the training set. These 36 patients were replaced with 20 colorectal cancer cases and 16 normal controls from the training set, ensuring the new validation cohort remained the same size (N=72). The SignaL score threshold was determined as described using the new training set. In this exploratory experiment, we observed improved sensitivity for advanced adenomas, with SignaL detecting 10/20 advanced adenomas (50%, 95% CI: 29.9–70%) at 100% specificity (95% CI: 89.3–100%). Furthermore, all 20 colorectal cancer cases included in the new validation set were correctly classified as positive (100%, 95% CI: 83.3–100%). Supplementary Figure S5 illustrates the sorted scores of the 72 subjects evaluated in this analysis. While these results suggest that training on advanced adenoma samples could significantly enhance SignaL’s performance, we emphasize the limitation of this experiment, as it involves adenoma subjects sampled from the same cohort in the training and validation sets.

References:

1. M. Mohri, A. Rostamizadeh, A. Talwalkar, *Foundations of machine learning*. (MIT press, 2018).

2. H. Zou, T. Hastie, Regularization and variable selection via the elastic net. *Journal of the royal statistical society: series B (statistical methodology)* **67**, 301-320 (2005).

3. C. Douville *et al.*, Detection of aneuploidy in patients with cancer through amplification of long interspersed nucleotide elements (LINEs). *Proceedings of the National Academy of Sciences* **115**, 1871-1876 (2018).

4. C. Douville *et al.*, Assessing aneuploidy with repetitive element sequencing. *Proc Natl Acad Sci U S A* **117**, 4858-4863 (2020).

5. C. Terao *et al.*, Chromosomal alterations among age-related haematopoietic clones in Japan. *Nature* **584**, 130-135 (2020).

6. R. Saiki *et al.*, Combined landscape of single-nucleotide variants and copy number alterations in clonal hematopoiesis. *Nature Medicine* **27**, 1239-1249 (2021).

7. P.-R. Loh *et al.*, Insights into clonal haematopoiesis from 8,342 mosaic chromosomal alterations. *Nature* **559**, 350-355 (2018).

8. H. Zheng, M. S. Zhu, Y. Liu, FinaleDB: a browser and database of cell-free DNA fragmentation patterns. *Bioinformatics* **37**, 2502-2503 (2021).

9. R. Elston, On Fisher's method of combining p‐values. *Biometrical journal* **33**, 339-345 (1991).
