# Supplementary figures and images for: A Blood-Based Assay for Detection of Patients with Advanced Adenomas
Source: Cancer Res Commun. 2025 Apr 16;5(4):621–31. doi: 10.1158/2767-9764.CRC-24-0398 (PMC12001750; doi:10.1158/2767-9764.CRC-24-0398)

## Slide 1
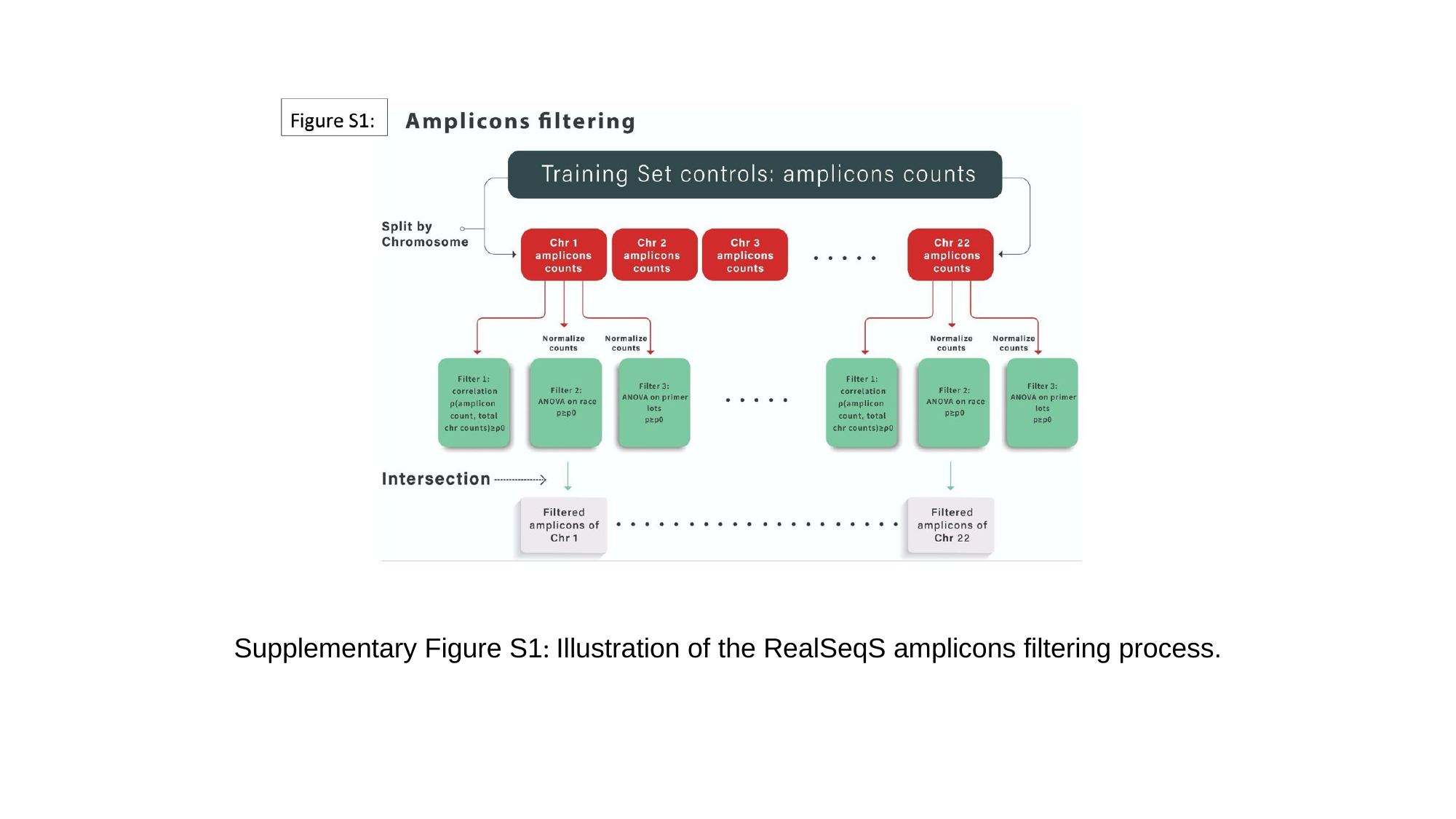

Supplementary Figure S1: Illustration of the RealSeqS amplicons filtering process.

Supplement: Figure S1 — Supplementary Figure 1: Illustration of the RealSeqS amplicons filtering process. [file crc-24-0398_figure_s1_suppsf1.pptx]

## Slide 1
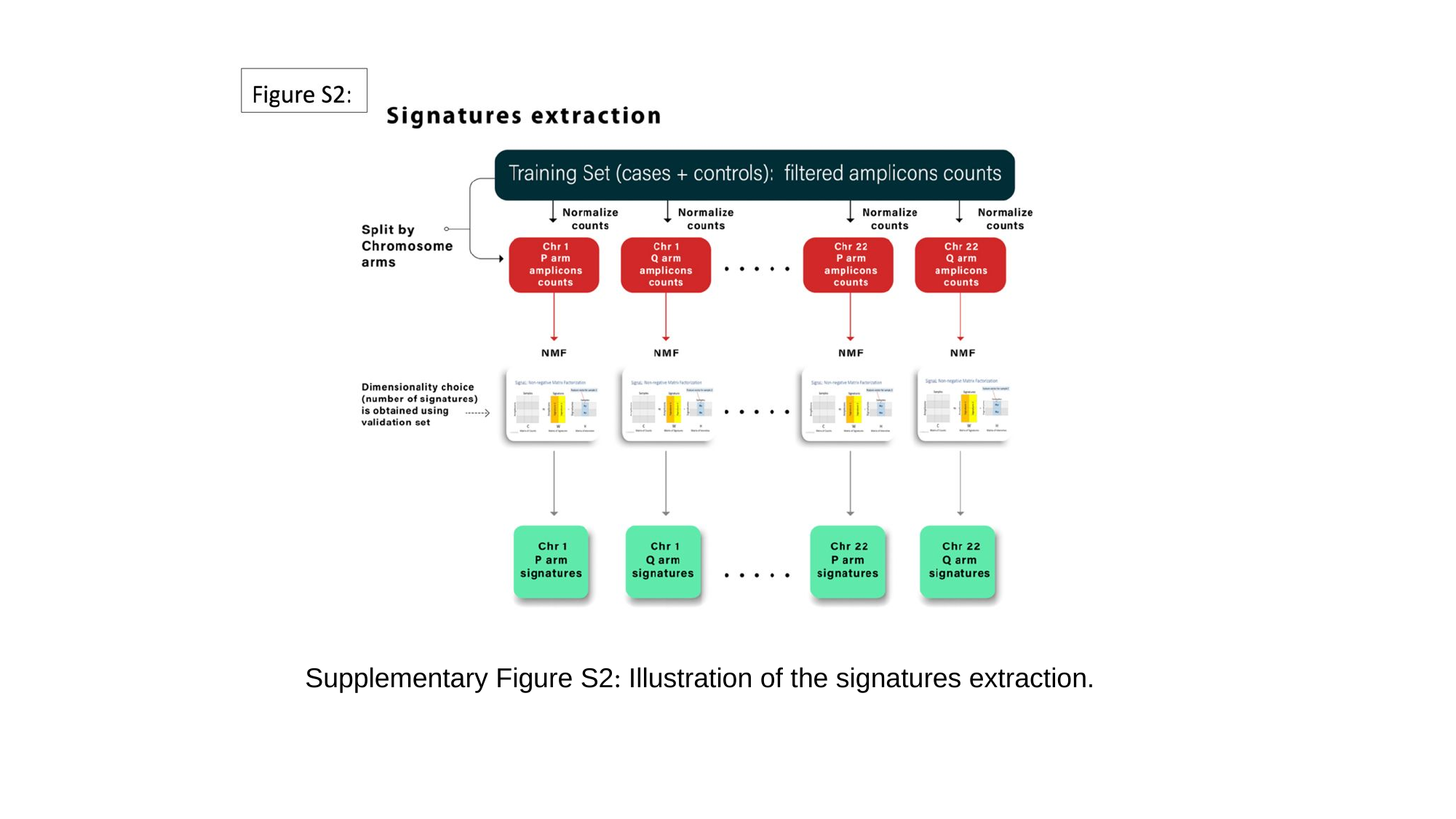

Supplementary Figure S2: Illustration of the signatures extraction.

Supplement: Figure S2 — Supplementary Figure S2: Illustration of the signatures extraction. [file crc-24-0398_figure_s2_suppsf2.pptx]
