# Supplementary material for: A Blood-Based Assay for Detection of Patients with Advanced Adenomas
Source: Cancer Res Commun. 2025 Apr 16;5(4):621–31. doi: 10.1158/2767-9764.CRC-24-0398 (PMC12001750; doi:10.1158/2767-9764.CRC-24-0398)
Supplement: Figure S3 — Supplementary Figure S3: Non-negative factorization of the counts matrices. Every chromosomal arm is represented by a matrix. Before factorization, the amplicon counts are normalized via dividing the raw counts by the total number of counts coming from each chromosomal arm. [file crc-24-0398_figure_s3_suppsf3.pptx]

## Slide 1
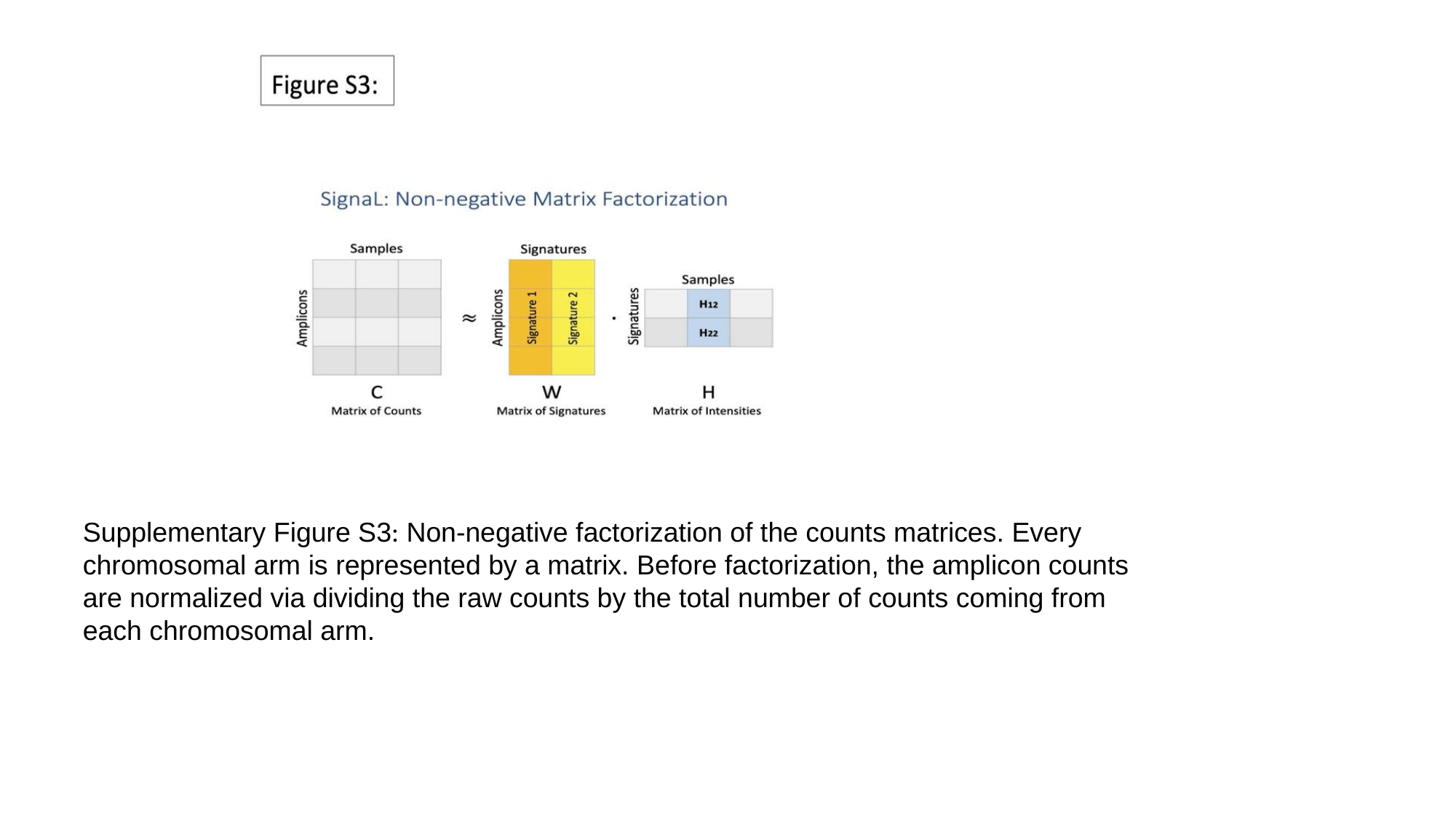

Supplementary Figure S3: Non-negative factorization of the counts matrices. Every chromosomal arm is represented by a matrix. Before factorization, the amplicon counts are normalized via dividing the raw counts by the total number of counts coming from each chromosomal arm.
