# Supplementary material for: A Blood-Based Assay for Detection of Patients with Advanced Adenomas
Source: Cancer Res Commun. 2025 Apr 16;5(4):621–31. doi: 10.1158/2767-9764.CRC-24-0398 (PMC12001750; doi:10.1158/2767-9764.CRC-24-0398)
Supplement: Figure S4 — Supplementary Figure S4: Illustration of the SignaL features extraction and selection procedure. [file crc-24-0398_figure_s4_suppsf4.pptx]

## Slide 1
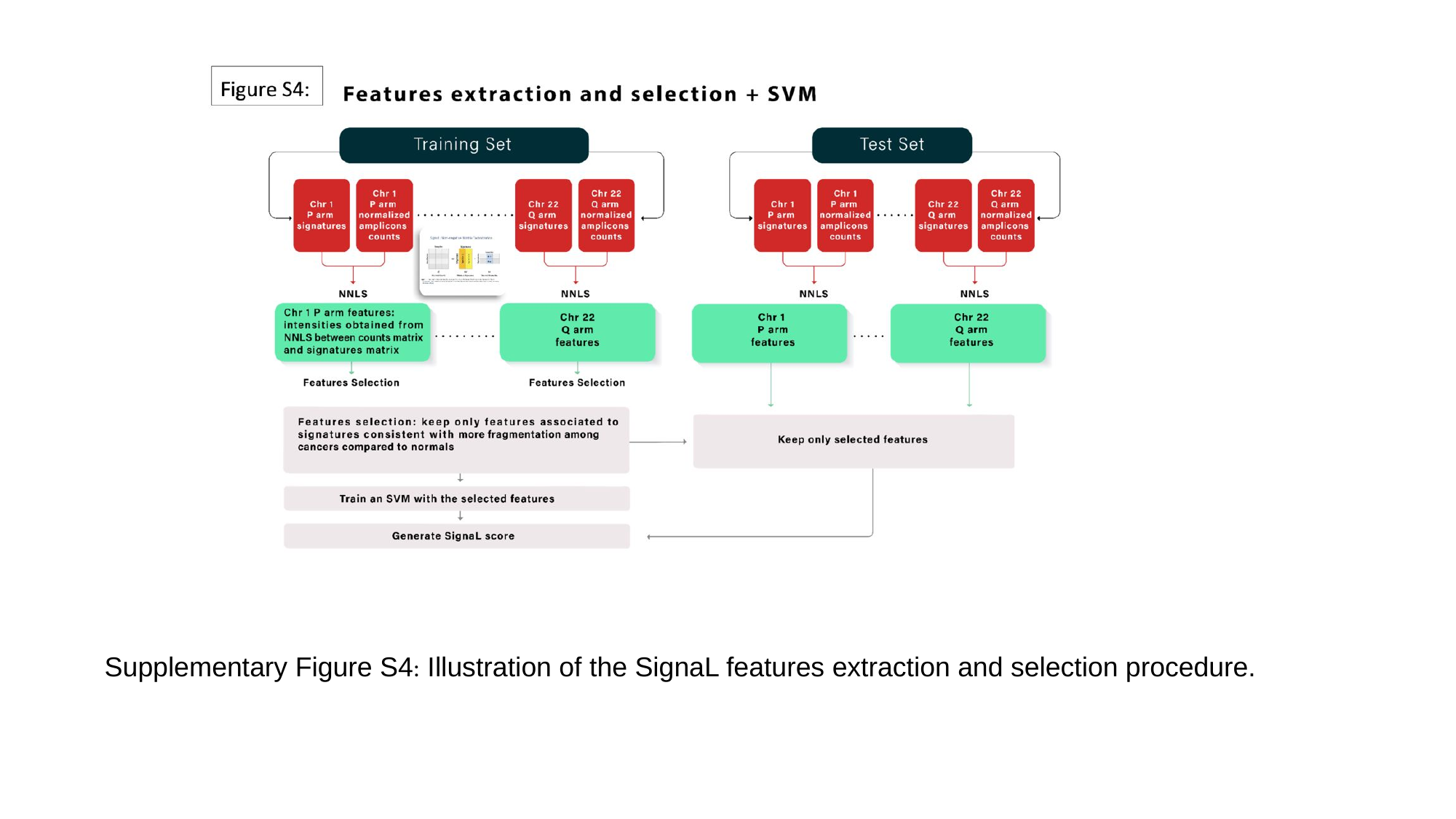

Supplementary Figure S4: Illustration of the SignaL features extraction and selection procedure.
