# Supplementary material for: A Blood-Based Assay for Detection of Patients with Advanced Adenomas
Source: Cancer Res Commun. 2025 Apr 16;5(4):621–31. doi: 10.1158/2767-9764.CRC-24-0398 (PMC12001750; doi:10.1158/2767-9764.CRC-24-0398)
Supplement: Figure S5 — Supplementary Figure S5: Sorted SignaL scores in plasma from 20 subjects with advanced adenomas, 20 subjects with colorectal cancer, and 32 control subjects, after including 20 subjects with advanced adenoma in the training set. [file crc-24-0398_figure_s5_suppsf5.pptx]

## Slide 1
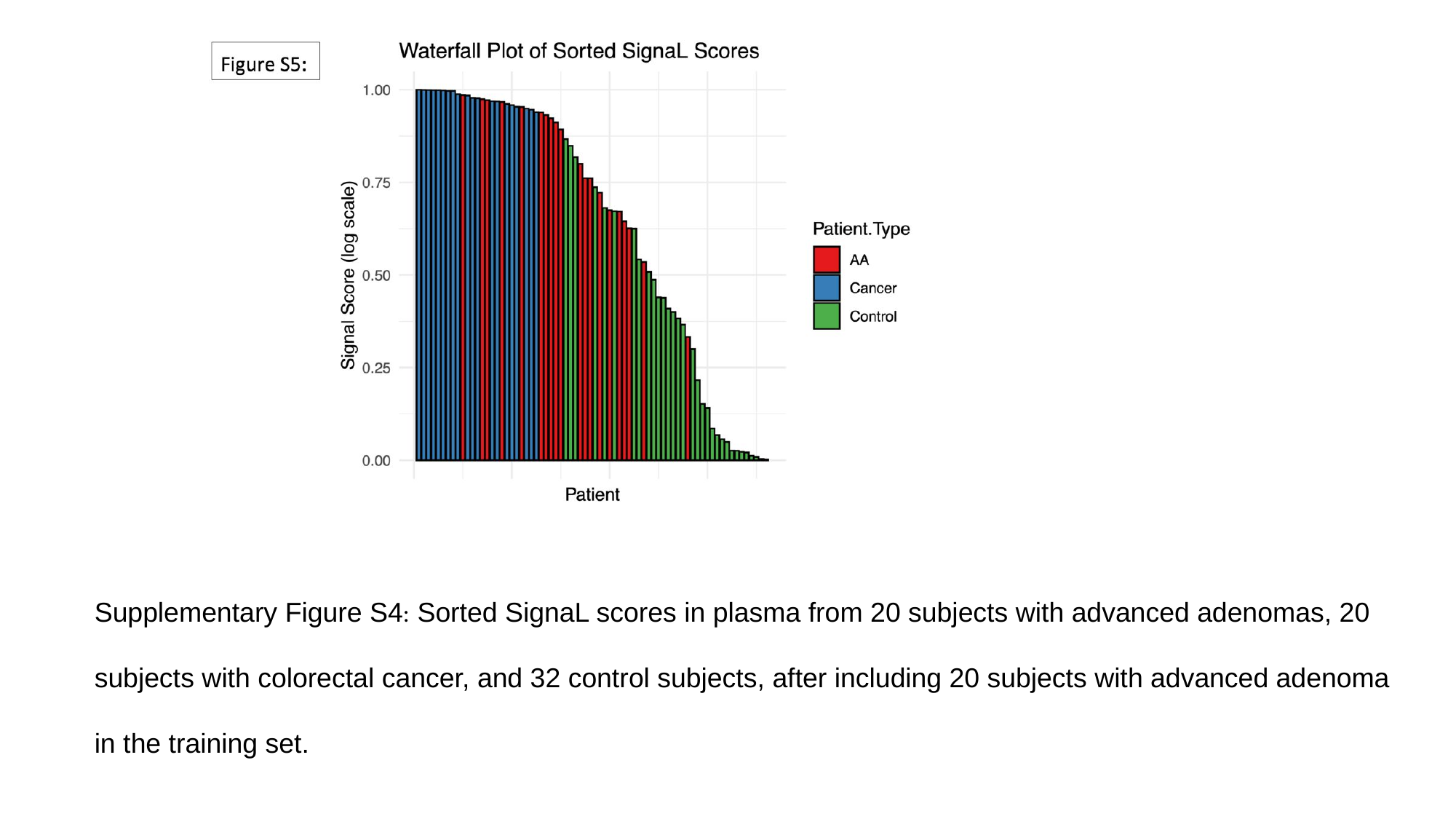

Supplementary Figure S4: Sorted SignaL scores in plasma from 20 subjects with advanced adenomas, 20 subjects with colorectal cancer, and 32 control subjects, after including 20 subjects with advanced adenoma in the training set.
