# Supplementary material for: A Blood-Based Assay for Detection of Patients with Advanced Adenomas
Source: Cancer Res Commun. 2025 Apr 16;5(4):621–31. doi: 10.1158/2767-9764.CRC-24-0398 (PMC12001750; doi:10.1158/2767-9764.CRC-24-0398)
Supplement: Supplementary Data — Statistical Approach for Mutations [file crc-24-0398_supplementary_data_suppsd2.pdf]

Methods for evaluating MAF significance

1 Setting and principles of the method

In each patient, 10 ml of plasma are taken at one time point after surgery. The DNA extracted from plasma is then divided into 95 wells, and a Polymerase Chain Reaction (PCR) is performed in each well. We would like to assess the presence of certain mutations of interest in a patient. The mutations that we test are the mutations that are observed in the primary tumor of that patient. In each well, and for every mutation we estimate the initial proportion of mutant DNA present, by computing the Mutant Allele Frequency (MAF) at that position. The MAF is defined as usual (ref.) by the number of super mutants (SMs) divided by the number of unique identifiers (UIDs). Therefore, every mutation is assessed using 95 measurements corresponding to the 95 wells observed. Even if the mutations that we would like to assess are the ones observed in the primary tumor, we also measure the MAFs of all mutations present in the amplicons containing the mutations of interest. These other mutations will be used as control mutations when computing the significance of our score as described further. Finally, the same experiment is run on a normal plasma that we use as a control template. Notice that we have 2 types of measurements that we are using as controls: the MAFs of mutations that are not present in the primary tumor but are found on the amplicons from the patient's plasma, and the MAFs of all (including the ones present in the primary tumor) from the control normal plasma. The general steps for obtaining an overall patient score and statistical significance are the following:

1. For every mutation of interest, score every well according to the observed MAF for that well. The score will be obtained after comparing that MAF with the MAFs observed in the control plasma but also taking into account the (estimated) number of genome equivalents in the considered well.

2. For every mutation of interest, combine all wells' scores in order to get an overall score for that mutation. The significance of that score will be obtained after comparing it with the overall scores of control mutations from the patient plasma.

3. Combine the scores of all the mutations to get a final score for the patient (p-value).

2 Scoring a single well for a fixed mutation

In an individual well, we observe an MAF for the considered mutation. Our score is based on 2 comparisons :

1. Compute a p-value associated to the observed MAF. For that, the null distribution is built using the MAFs observed in the control (normal) plasma. We will call this null distribution, the first null.

2. Given an estimation of the number of Genome Equivalents (GE), and assuming that we started with $\frac{1}{GE}$ mutants, decide whether the MAF observed is plausible or too low for an initial signal equal to $\frac{1}{GE}$. For this part, we will construct another null distribution as an additional sanity check. We will call this other null distribution the second null distribution. Notice that what we call the second null distribution is the closest alternative to the null.

2.1 Building the first null distribution using the control plasma

Let the set of mutations considered in the control plasma be denoted by $m_1, m_2, ...m_N$. Recall that this set is not just the mutations observed in the primary tumor, but all mutations found in the considered amplicons. In theory, if we have an amplicon of size l, and we consider all the possible types of mutations in the amplicon, the total number of possible mutations N grows exponentially (considering possible insertions and deletions). However, we bound the possible number of mutations by the maximum between the following numbers:

- The number of mutations observed in that amplicon, in the patient plasma or the control plasma.

- $3l$, which is the number of possible mutations that are substitutions.

The number of data points observed for building the control distribution can be summarized in a matrix of size $N \times N_{Wells}$, where N_{Wells} is the number of wells. Each row i corresponds to the N_{Wells} measurements of MAF obtained after PCR for mutation m_i. Each MAF measurement can be 0 or some positive number.

For every mutation m_i, the null distribution is modeled by a mixture of a delta at 0 and a continuous distribution. More precisely: the $log(MAF)$ for mutation m_i is modeled by a mixture of a delta at $-\infty$ and a mixture of K Gaussians:

$$f_i(x) = p_i \delta_{-\infty}(x) + (1 - p_i) \sum_{k=1}^{K} w_{i,k} \mathcal{N}(\mu_k, \sigma_k)(x), \quad (1)$$

where:

- $f_i(x)$ is the density of $log(MAF)$ for mutation m_i.
- p_i is the probability of observing a zero MAF (i.e $\log(MAF) = -\infty$). Notice that this probability is mutation dependent, meaning that in our model, the probability of observing an MAF under the null distribution varies from one mutation to another. This assumption is made because we know that some mutations are more likely than others to be observed via PCR errors.
- $w_{i,k}$ are the weights of each Gaussian in the mixture . Notice that these weights are mutation dependent for the same reason stated above.
- μ_k and σ_k are the means and variances of the Gaussians in the mixture. These parameters are the same for all mutations.
- K is the number of Gaussians in the mixture.
- The estimation of the number of components K is based on a mathematical model of the PCR process.
- The estimation of the p_i's is based on a Bayesian Beta-Binomial model, where the parameters of the Beta are shared across all mutations.
- The estimation of the weights ($w_{i,k}$), the means and variances of the Gaussians is based on an Expectation Maximization (EM) algorithm.

2.1.1 Estimation of the p_i's

We recall that the number of wells is denoted by N_{Wells} and we are considering N mutations $m_1, ..., m_N$ in the control plasma. For every i, let us denote by Z_i the number of wells among N_{Wells} where we observe a zero MAF. To estimate p_i, we assume the following model. For every i, given p_i, Z_i is a binomial:

$$Z_i | p_i \sim Bin(p_i, N_{Wells}), \tag{2}$$

where the Z_i's are independent given the p_i's. The p_i's are realizations of i.i.d random variables:

$$p_i \sim Beta(a, b), \tag{3}$$

for some parameters a and b. We estimate a and b by using the laws of total expected values and variance to get the method of moments estimators for a and b. Finally, the estimators $\hat{p}_i$ are obtained by considering the posterior values from the beta-binomial:

$$\hat{p}_i = \mathbb{E}(p_i | Z_i, a, b) = \frac{Z_i + a}{a + b + N_{Wells}} \tag{4}$$

2.1.2 Estimation of K

If one considers a PCR experiment with 1 GE, the possible positive $MAFs$ that can be observed, up to a first order approximation, are of the form 2^{-i} where $i \in \{1, 2, 3, ..., N_{cycles}\}$, where N_{cycles} is the number of PCR cycles. For each i, an MAF of the form 2^{-i} is obtained (up to a first order approximation which assumes PCR is efficient) with probability $\mu 2^{i-1}$, where μ is the mutation rate (probability of a mutation per DNA duplication) at a specific position via PCR error (see Figure 1).

Therefore, the probability of observing an MAF of the form 2^{-i}, given that the MAF is non zero (a conditional probability), should be:

$$\frac{2^{i-1}}{\sum_{l=1}^{N_{cycles}} 2^{l-1}}. \tag{5}$$

Note that this probability does not depend on μ. Now considering any number of GEs, $|GE|$, the possible MAFs will be of the form $\frac{2^{-i}}{|GE|}$ with the same probabilities of equation 5. Now, let n be the number of wells having positive MAFs in the control plasma for that particular mutation. If we denote by p_i the probability in equation 5, then the expected number of different $MAFs$ observed in an experiment can be estimated by:

$$\sum_{i=1}^{N_{cycles}} \left(1 - (1 - p_i)^n\right). \tag{6}$$

This expected number almost gives us our estimator of the number of components K up to a last detail: we merge together $MAFs$ that are 10^{-4} close to each others.

2.1.3 Estimation of the weights, means and variances of the mixture of Gaussians)

As mentioned above, we take an EM approach to estimate the parameters of the mixture. More precisely, denote by $x_{i,j}$, $1 \leq j \leq n_i$ the logs of positive $MAFs$ observed for mutation m_i. Notice that n_i obviously depends on the mutation. If n_i is 0, the mutation is not used for the estimation of the mixture's parameters. Denote by N_o the number of mutations where at least one positive MAF was observed. We assume that each observation $x_{i,j}$ is associated with a hidden random variable $\xi_{i,j}$ taking values in $1, ..., K$ and corresponding to the Gaussian of the mixture that generated $x_{i,j}$. Notice that the EM approach here is different from the usual one used for estimating the parameters of a mixture of Gaussians as the weights of the mixture are different for every mutation but the means and variances are shared among the mutations. However, we use the regular Gaussian mixture model where we merge all mutations together for the initialization of the weights, means and variances estimators. After the initialization, the iterations of the EM algorithm are as follows. Given the parameters, compute for every i, j, k:

$$\mathbb{P}\left(\xi_{i,j} = k | x_{i,j}, w_{i,k}, \sigma_k^2, \mu_k\right) = \frac{w_{i,k} f_k(x_{i,j})}{\sum_{k=1}^K w_{i,k} f_k(x_{i,j})}, \text{(E step)}, \tag{7}$$

where f_k is the density of a Gaussian with mean μ_k and variance σ_k^2. Then, given $p_{i,j,k} := \mathbb{P}\left(\xi_{i,j} = k | x_{i,j}, w_{i,k}, \sigma_k^2, mu_k\right)$, update:

$$w_{i,k} = \frac{1}{n_i} \sum_{j=1}^{n_i} p_{i,j,k}, \text{(M step)}, \tag{8}$$

$$\mu_k = \frac{1}{n_i N_o} \sum_{i=1}^{N_o} \sum_{j=1}^{n_i} p_{i,j,k} x_{i,j}, \text{(M step)}, \tag{9}$$

$$\sigma_k^2 = \frac{1}{n_i N_o} \sum_{i=1}^{N_o} \sum_{j=1}^{n_i} p_{i,j,k} (x_{i,j} - \mu_k)^2, \text{(M step)}. \tag{10}$$

We alternate between the E step and M step until convergence of the parameters. Finally, if a mutation does not have any well with a positive MAF, the corresponding weights $w_{i,k}$ of the mutation are simply those of the initialization step, i.e, the global weights of the mixture obtained after merging all mutations together. This ends the description of the parameters estimation procedure used for building the null distribution.

2.1.4 Some qualitative remarks about the estimator of the first null distribution

In general, the higher the mutation's PCR error probability, the lower is the weight of the point mass at 0 and the heavier are the tails of the continuous

density part (see Figures 2 and 3). However, if we don't observe an $MAF > 0$ for a particular mutation in the control templates, we do not put 100% of the mass on 0 in our corresponding control distribution. We still have a continuous part (see Figure 4). The reason is the following: The estimated control distribution is a mix between a distribution using only the mutation specific MAFs of the controls and a global null using all mutations MAFs of the control templates. The more positive MAFs we observe in the controls for a particular mutation the smaller the weight we put on the global distribution and vice versa.

2.2 Building the second null distribution

The idea is the following. Assume that we start PCR with a given number of genome equivalents that we denote by GE. If there was a mutation already present, the lowest initial MAF before PCR is $\frac{1}{GE}$. We would like to assess whether or not the observed final MAF is plausible for an initial signal of $\frac{1}{GE}$. For this, we use some "spike-in" experiments as control to learn the control distribution of the MAFs in the situation where we start with only 1 mutant out of GE template. More precisely, we used 8 experiments where we could control the number of GE per well and the average number of mutants per well. These numbers are as follows:

Experiment ID	Initial nb of GEs per well	Avg nb of mutants/well
6881	376	0.383
6923	376	0.383
6961	376	0.383
6924	376	0.383
6963	602	0.383
6960	376	0.383
6882	376	0.383
7049	376	0.383

Contrary to the first null distribution, here we do not differentiate between mutations, i.e. the second null distribution is mutation independent.

2.2.1 Statistical model

We will consider a well where we start with 376 GEs per well and 1 mutant as our "base" case. We assume that the number of UIDs obtained per well when we start with 376 GEs per well after PCR follows a negative binomial $nb(r_1, p_1)$ where r_1 is the size parameter and p_1 is the parameter of success. In general, when we start with any number GE of genome equivalents, the number of UIDs obtained per well follows a negative binomial $nb(\frac{G}{376}r_1, p_1)$. We assume that when we start with 1 mutant per well , the number of super mutants (SM) obtained per well (given that the $MAF > 0$) follows a negative binomial $nb(r_2, p_2)$ where r_2 is the size parameter and p_2 is the parameter of success. In general, when we start with a number M of mutants, the number SM that is obtained per well follows a negative binomial $nb(M \times r_2, p_2)$. Finally, we need to model the dependence between the number of SMs and the number of UIDs as they are clearly correlated. For this, we use a Gaussian copula model. More precisely, if we denote by $\boldsymbol{\phi_\rho}$ the bivariate C.D.F of a bivariate gaussian with variances equal to one and a covariance that is equal to ρ, by ϕ the CDF of a standard Gaussian and $F_{r,p}$ the C.D.F of a negative binomial $nb(r, p)$, then the bivariate C.D.F of SM and $UIDs$, that we denote by ψ is nothing but:

$$\psi(k_1, k_2) = \boldsymbol{\phi_\rho}\left(\phi^{-1} \circ F_{r_1,p_1}(k_1), \phi^{-1} \circ F_{r_2,p_2}(k_2)\right). \tag{11}$$

Remark: for this particular point, to avoid problems that might arise due to the fact that the negative binomial is a discrete distribution and that the CDF is not exactly a uniform on $[0, 1]$, we consider that the copula model is on $k_2 + U$ and $k_2 + U$ where U is an independent uniform random variable on $[0, 1]$.
Hence if we estimate the parameters of this model and since $MAF = \frac{SM}{UID}$, if we have a well with GE genome equivalents and 1 initial mutant, then we have the full distribution of the MAF.

2.2.2 Estimation of the parameters of the model

The parameters to estimate are r_1, p_1, r_2, p_2 and the parameter ρ driving the correlation between SM and UID. Our set of observations consists of all the

positive MAFs observed in every well of all 8 experiments. Notice that we know the initial number of GEs for every well in these experiments. However, we know the initial average number of mutants per well but for every well the number of mutants M is in fact a hidden variable. Since we know that the average initial number of mutants per well is 0.383, we assume, for our training set (observations), that the number of initial mutants per well given that $maf > 0$ in that well follows a Poisson distribution with intensity parameter 0.383 conditioned on the event that the Poisson is positive. Also, given the number of super mutants SMs, we assume that the initial number of mutants are independent across wells. To estimate M:

- For each well, we initialize M to M_0. We then estimate r_2, p_2 using the method if moments.
- Now, given r_2, p_2, we estimate M as the initial number of mutants giving the maximum a posteriori probability (given SM).
- Using the estimated M's we recompute r_2, p_2 using the method of moments.
- We repeat the procedure until convergence of the M's.

Once estimated, the M's are at this point assumed to be the observed initial number of mutants per well. Finally, having for every well and all experiments the initial number of GEs and mutants, we estimate the parameters r_1, p_1, r_2 and p_2 using the training set via the method of moments. We do the same for the parameter ρ that is driving the correlation between SM and $UIDs$.

2.3 Scoring a particular mutation in a single well using the two null distributions

Recall that our first goal is to score a particular mutation m_i from the patient plasma in a single well using the observed MAF of the mutation. Denote by $x_{i,j}$ the logarithm of the observed MAF (it is $-\infty$ if the $MAF = 0$).

The first step to compute the score is the following. Using the first null distribution, we can compute a corresponding p-value:

$$\mathbb{P}_{00}(X > x_{i,j}) = (1 - p_i) \sum_{k=1}^{K} w_{i,k} F_k(x_{i,j}), \qquad (12)$$

where the probability $\mathbb{P}_{00}$ corresponds to the probability under the first null distribution and $F_k(x)$ is the probability that a Gaussian with mean μ_k and variance σ_k^2 is larger than x. Taking minus the logarithm of the p-value that we denote by $p_{i,j}$, we get a first intermediary score, $-\log(p_{i,j})$. The second step uses the second null distribution in the following way. Denote by $\mathbb{P}_{01}$ the probability under the second null distribution. Fix a certain tolerance level that in our case we set to be 5×10^{-3}. We can compute the threshold $t(GE)$ such that:

$$\mathbb{P}_{01}(\log(MAF) < t(GE)|MAF < \frac{1}{GE}) = 5 \times 10^{-3}, \quad (13)$$

where GE is the number of genome equivalents associated to the observed MAF. We are now ready to define the score $s_{i,j}$ of mutation in a single well:

$$s_{i,j} = -\log(p_{i,j}) \times \mathbf{1}_{p_{i,j} \leq 0.05} \times \mathbf{1}_{\log(p_{i,j}) > t(GE)}, \quad (14)$$

where $\mathbf{1}$ is the indicator function. In other words, the score s_{ij} is given by the negative of the logarithm of the p-value of the MAF associated to the first null distribution, if the MAF is high enough, and zero if the MAF does not meet the requirements that its associated p-value is not higher than 0.05, while at the same time it must be large enough to pass the second test associated to the second null distribution. We have thus defined the score of a particular mutation in a particular well. If an MAF passes the tests for a particular mutation, we say that the well is positive for that mutation.

3 Overall score of a mutation

Recall that we have a set of mutations in the patient's plasma that we would like to test. These mutations are those observed in the primary tumor. We look at all these mutations in the considered amplicons. We will use those that were not in the primary tumor as control mutations. More precisely, denote by I_0 the index of all mutations that are control mutations and I_1 the set of mutations that were present in the patient plasma. For every i, whether it is in the control set or not, we can attribute an intermediary score:

$$s_i := \sum_{j=1}^{N_{Wells}} s_{i,j}. \quad (15)$$

To get the final score of a mutation, we associate a p-value $\tilde{s}_i$ to s_i in the following way:

$$\tilde{s}_i = \frac{1}{|I_0|} \sum_{l \in I_0} \mathbf{1}_{s_l > s_i} \quad (16)$$

4 Scoring the patient

We are finally ready to define the overall score of a given patient denoted by S:

$$S := \sum_{i \in I_1} -2\log(\tilde{s}_i) \quad (17)$$

To associate a p-value to the score S we simply compare it to a $\chi^2(2|I_1|)$ distribution.
